# Impacts of Surface Reconstruction and Metal Dissolution on Ru_1–*x*_Ti_*x*_O_2_ Acidic Oxygen Evolution Electrocatalysts

**DOI:** 10.1021/acs.jpcc.4c08119

**Published:** 2025-02-10

**Authors:** Francisco A. Ospina-Acevedo, José Fernando Godínez-Salomón, Zachary G. Naymik, Kevin C. Matthews, Jamie H. Warner, Christopher P. Rhodes, Perla B. Balbuena

**Affiliations:** † Department of Chemical Engineering, Texas A&M University, College Station, Texas 77843, United States; ‡ Department of Chemistry and Biochemistry, Texas State University, San Marcos, Texas 78666, United States; § Materials Science, Engineering and Commercialization Program, Texas State University, San Marcos, Texas 78666, United States; ∥ Texas Materials Institute, The University of Texas at Austin, Austin, Texas 78712, United States; ⊥ Walker Department of Mechanical Engineering, The University of Texas at Austin, Austin, Texas 78712, United States

## Abstract

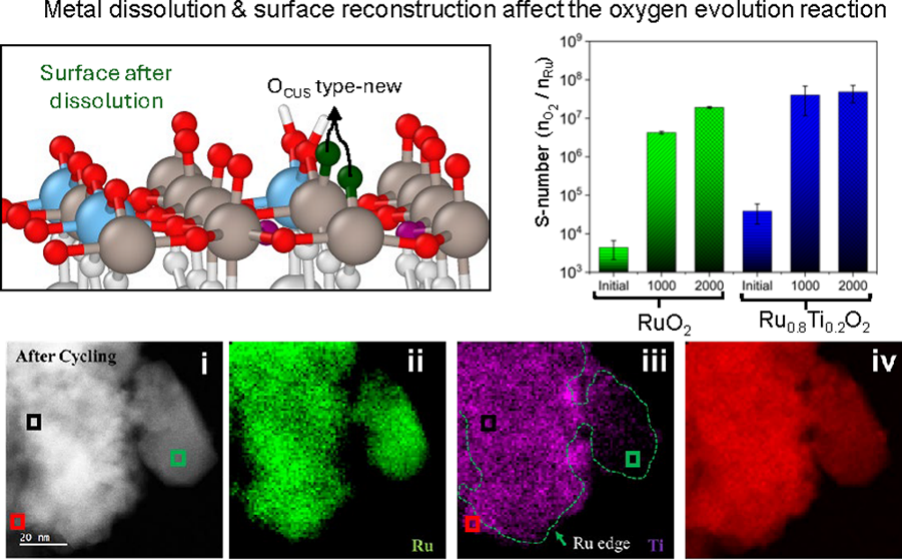

Improved oxygen evolution reaction (OER) electrocatalysts based on an additional understanding of surface changes that occur upon metal dissolution are needed to enable the efficient use of electrochemical water splitting. This work integrates theoretical and experimental studies of the effects of metal dissolution from the RuO_2_ and Ru_1–*x*_Ti_*x*_O_2_ surfaces on the OER activity and electrochemical stability. Our computational analysis shows that the energetic barriers for metal dissolution depend highly on the surface site and Ti-substituent location. Metal dissolution induces the formation of new active surface sites with different electronic density distributions. In addition to dissolution-induced changes to the surface composition, electron density changes occur in the interfacial electrolyte components. Surface reconstruction changes the activation barriers for the OER steps. Our experimental analysis of RuO_2_ and Ru_0.8_Ti_0.2_O_2_ using a two-step durability test in acidic electrolytes shows that the OER activity, surface, and metal dissolution change over the durability tests. Ti-substitution exhibits improved electrochemical stability with cycling. For RuO_2_, changes in the mass activity of RuO_2_ with cycling are directly correlated with Ru dissolution and lowering of the electrochemical surface area (ECSA). In contrast, Ru_0.8_Ti_0.2_O_2_ showed a 19 times lower Ru dissolution rate, and metal dissolution results in increasing the ECSA and new active sites. Our STEM and EELS analysis supports that repeated cycling under OER conditions results in surface reconstruction for both RuO_2_ and Ru_0.8_Ti_0.2_O_2_, with the formation of a disordered RuO_2_ surface and changes to the distribution of Ru and Ti at the Ru_0.8_Ti_0.2_O_2_ surface. The experimentally observed changes in activity and surface structure after cycling are consistent with computational analysis, which shows how metal dissolution may alter the OER activation barriers. Combining experimental and computational insights, this work reveals the effects of metal dissolution on the surface atomic and electronic structure and OER activity and advances our comprehension of metal dissolution dynamics and surface reconstruction, which may have implications for other catalytic processes.

## 1 Introduction

The electrochemical splitting of water is a promising way to store renewable energy in the form of H_2_ fuel in an environmentally friendly manner.^1−3^ Noble metal-based catalysts, such as RuO_2_ and IrO_2_, are currently regarded as the best candidates for the oxygen evolution reaction (OER) at the anode in proton exchange membrane (PEM) electrolyzers.^4−10^ However, the acidic operating conditions of these devices pose a challenge for OER due to the rapid degradation of even noble metal-based catalysts.^3,9,11,12^ Therefore, developing highly active, stable, and cost-effective OER electrocatalysts is crucial for the widespread use of PEM electrolyzers.^3,13,14^ Although ruthenium-based catalysts exhibit high acidic OER activity with low energy input compared to other materials,^12,15,16^ they are unstable under PEM electrolyzer operating conditions and exhibit significant catalyst degradation.^12,17,18^

Pure and mixed metal oxides have been investigated to improve the activity and stability of OER electrocatalysts, where the substituent or dopant species may improve the performance compared to the base material.^19−24^ Theoretical work has investigated pure metal oxides (RuO_2_,^9,25^ and IrO_2_ and TiO_2_^26^) initial OER and metal dissolution. Building from pure metal oxides, a significant need is to understand how metal substituents affect surface reconstruction, activity, reaction mechanisms, and dissolution and determine interrelationships between these multiple, dynamic processes that occur within similar time scales. Accurately evaluating mass activity in mixed metal oxides is complicated by atypical surface metal compositions, even though it is expected that the total mass activity of the mixed metal oxide compound will be lower as less active oxides are added.^27^ Our groups^28−32^ and others^33−40^ have conducted metal substitution studies to improve OER electrocatalyst performance. Most of these efforts focus on understanding the effects of the catalyst activity. Recent studies report how the surface is reconstructed and how OER activity changes following electrochemical stability tests that show dissolution occurs.^26,41−49^ Dissolution measurements show that the OER electrocatalyst surface is dynamic,^50^ which is contrary to conventional thinking that electrochemical interfaces are static environments. The OER mechanism and kinetics also rely on the surface’s structural evolution under dynamic operation conditions, and such surface reconstruction is expected to influence both OER and metal dissolution.^9,51−56^ Well-defined RuO_2_ surfaces showed an initially higher Ru corrosion rate and a decreased Ru corrosion rate upon a second stability test, suggesting that the surface may be reorganized following dissolution and reach a more steady state.^49^

Oxygen evolution electrocatalysts like Ru, Ir, and Pt have been shown to exhibit an inverse relationship between activity and electrochemical stability.^11,12,15,57,58^ Still, there is no consensus on how these processes are related.^59,60^ The lack of information on the activity-stability-morphology relationships hinders understanding of whether OER and catalyst dissolution share common reaction intermediates and how these are affected by catalyst morphology.^49,61^ Hence, mechanistic information is needed to understand the degree of interaction, coupling, or decoupling between these two processes to design electrocatalysts with enhanced OER activity while minimizing electrode dissolution, ideally with atomic-scale precision.^52,62^ In addition, improvements in OER activity have been achieved through defect engineering and metal doping by understanding the formation of highly reactive surface species during catalyst degradation.^9,63−67^ Such information is crucial for the activity and stability. Still, experimental probing of electrocatalyst/water interfaces at the nanoscale to detect short-lived reaction intermediates is challenging, limiting mechanistic investigations of electrocatalyst corrosion.^9^ Thus, there is a clear need to understand the catalyst surface evolution further and the changes arising on the surface due to the competition between the OER and the degradation reactions.

We recently investigated substituent effects on Ru_1–*x*_M_*x*_O_2_(110) (M = Ti, Zr, Nb, Ta) surface activity and OER mechanism and performed a preliminary analysis of metal dissolution for active metal and substituents.^29,68^ We concluded that Ti and Zr substituents at specific sites yield higher theoretical OER activity than RuO_2_, with Zr substitution suggesting an alternative OER mechanism. At the same time, the effects on electrochemical stability via preliminary dissolution analyses showed increased activation barriers for Ru dissolution compared with the base RuO_2_ electrocatalyst. Here, we report a coupled theoretical and experimental approach to characterize the effects of metal dissolution from RuO_2_ and Ru_1–*x*_Ti_*x*_O_2_ on surface reconstruction, OER activity, and electrochemical stability. We evaluate possible correlations between metal incorporation and metal–electrolyte interactions that lead to metal dissolution. We then examine surface reconstruction and subsequent formation of new active sites and compare surface reactivity before and after dissolution. We use theoretical and experimental techniques to carry out the proposed analyses. The thermodynamic integration method within the Blue Moon ensemble technique based on constrained ab initio molecular dynamics (c-AIMD) allows the evaluation of the free energy profile of specific events along a reaction coordinate,^69,70^ revealing the metal ion dynamic trajectory and energetic barriers for dissolution into the aqueous media. Although the c-AIMD technique does not have any explicit potential dependence, it allows the evaluation of energetic barriers for certain processes such as metal dissolution. We note that such an energetic barrier needs to be overcome for the process to take place, and external voltage can certainly be such a suitable energy source. Thus, although an applied voltage may also modify the surface and interfacial electrolyte structure, the calculated barrier should give us an estimate of the voltage needed to overcome it. Subsequent DFT and AIMD studies allow the characterization of the structural and chemical surface evolution. Experimentally, we tested RuO_2_ and Ru_0.8_Ti_0.2_O_2_ catalysts using a two-step accelerated durability test that utilized high potentials (1.85 V), reflective of the operating conditions of PEM electrolyzers. This test was coupled with measurements of Ru dissolution and microscopic and spectroscopic analyses of the materials before and after cycling to enable correlations between the OER activity, surface changes, and metal dissolution.

## 2 Methods

### 2.1 Computational Methods

All calculations are performed using the density functional theory (DFT) method as implemented in the Vienna Ab Initio Simulation Package (VASP – version 5.4.4),^71−73^ with the plane-wave basis set, including spin polarization, to describe the valence electrons using a cutoff energy of 450 eV. The treatment of the core–electron interactions is done with the recommended projector augmented wave pseudopotentials (PAW),^74,75^ i.e., Ru_pv, Ti_sv, O, and H. We use the revised Perdew–Burke–Ernzerhof generalized gradient approximation (revPBE-GGA),^76,77^ the exchange-correlation functional that has shown improvement in the energetics of adsorption phenomena on transition-metal surfaces.^78^ The energies and forces convergence criteria are set to 10^–5^ eV and 0.02 eV/Å, respectively, while long-range van der Waals interactions were incorporated using Grimme’s D3-dispersion correction.^79,80^ Inclusion of the Hubbard U correction as proposed by Dudarev et al.^81^ to the Ti revPBE-GGA functional is done. While many reported computational studies on RuO_2_ do not typically include Hubbard + U corrections, recent work has applied this correction to systems containing early and late transition metals, including RuO_2_, affecting their atomic and electronic structures.^82−84^ These studies found that the impact on RuO_2_’s structure and adsorption energies is small and the results follow trends obtained without U correction. So, although the use of U in LDA and GGA calculations is debated, its application to Ru does not appear essential for obtaining accurate results.^82−85^

On the other hand, the inclusion of Hubbard U, as proposed by Dudarev et al.^81^ to the Ti revPBE-GGA functional, addresses strong electron correlation in the partially filled d-shells of transition metal oxides. For Ti^4+^ species, self-interaction corrections are necessary due to the localized 3d and 4f orbitals, which complicate electronic structure descriptions.^86^ DFT+U corrects for d-orbital electron delocalization by adding a term to the Hamiltonian that increases the total energy when two electrons occupy the same cation.^87^ A wide range of U values has been reported for Ti, highlighting the system-dependent nature of this parameter,^86−108^ and several methods are available to calculate accurate U values.^88,109^ Since the optimal U parameter for Ti varies across systems, we adopted a commonly accepted approach, the linear response ansatz, as proposed by Cococcioni et al.,^109^ to calculate and apply a constant U value throughout all optimization and dynamic calculations. Details on the U value calculation are given in the Supporting Information of our previous work,^29^ and the value used in the present calculations is 5.6 (eV).

We use the continuum solvation model, implemented in the VASPsol code, for all ground-state optimizations.^110−112^ The Brillouin zone was integrated with the Gamma-centered Monkhorst–Pack grid method.^113^ The Gaussian smearing method with an energy smearing set to 0.02 eV allowed for the determination of electronic occupancies in all calculations. c-AIMD simulations, using the thermodynamic integration within the slow-growth approach as implemented in the Blue Moon ensemble method in VASP,^114−118^ were conducted using a single Gamma point for Brillouin zone integration, with a collective variable representing a metal displacement of 0.5 Å/ps to drive the dissolving metal (Ru or Ti) into the dissolved state. This collective variable was selected during the simulation to minimize the artificial interactions between the dissolving metal and the surrounding environment. Initially, the distance between the dissolving metal atom (M = Ru, Ti) and its nearest subsurface oxygen atom (O_sub_) was used to apply a force that pushed the metal atom from the surface into the aqueous phase. The collective variable was dynamically updated as the simulation progressed to maintain a consistent driving force. Specifically, once the metal atom moved sufficiently away from its original subsurface oxygen, the collective variable was adjusted to reflect the next nearest subsurface oxygen atom beneath the dissolving metal, ensuring that the applied force remained normal to the surface throughout the dissolution process. As shown in previous works,^9,119−122^ the displacement of 0.5 Å/ps is used as a compromise between simulation feasibility and physical realism.

The simulations, performed in the canonical ensemble, were temperature-controlled at 300 K using a Nosé–Hoover thermostat over the simulated time. The liquid phase was modeled with pure water, and 27 water molecules (forming around two monolayers) were placed on the surface using the BIOVIA Materials Studio packing module,^123^ targeting a density of 1 g/cm^3^. The mass of hydrogen was changed to that of tritium to safely use a time step of 1 fs. To prevent periodic boundary interactions in the *z*-direction, an optimized inert helium layer was positioned above the water packing at an optimized distance.^124,125^ Each intermediate step was equilibrated for 2 ps, and an additional 2 ps simulation was run to average the system’s force. The duration of each simulation depended on the appearance of the first stable dissolved species.

The RuO_2_ unit cell used to model the Ru_1–*x*_Ti_*x*_O_2_-(110) surfaces was taken from our previous work,^29^ whose lattice parameters are in excellent agreement with reported values.^126−129^ For these calculations, a fully oxidized, periodic 3-layer, 4 × 2-slab with a surface area of 12.43 × 12.68 Å^2^ and 10 Å of vacuum space on top of the surface was made to evaluate dissolution and activity after dissolution in the RuO_2_(110) and Ru_1–*X*_Ti_*X*_O_2_(110) surfaces, with the surface atomic concentrations of substituent species of 25 and 50 at %. For all calculations, the bottom layer atoms are fixed. Activation energy and transition state (TS) calculations are performed using the climbing-image nudged elastic band (CI-NEB) method,^130−132^ while the charge transfer is evaluated via the Bader charge analysis.^133−135^

### 2.2 Experimental Methods

#### 2.2.1 Synthesis and Structural Characterization

Ti-substituted ruthenium oxide Ru_0.8_Ti_0.2_O_2_ was synthesized following a hydrothermal method previously reported.^29^ Commercial RuO_2_ (Alfa Aesar, Product No. A10816.06; Lot No. 10237864) was used for comparison. Nitrogen physisorption measurements were obtained using a Micromeritics ASAP 2020 surface area and porosimetry analyzer. Samples were degassed under a vacuum at 120 °C for 16 h before analysis. Brunauer–Emmett–Teller (BET) surface areas were obtained from the nitrogen physisorption isotherms.

#### 2.2.2 Electrochemical Characterization

Electrochemical measurements were conducted at a constant temperature (298 K) in a three-electrode cell in 70 mL of 0.1 M HClO_4_ electrolyte (Veritas, 69–72%, 0.00001% chloride). The electrochemical data were collected with an Autolab PGSTAT128N bipotentiostat and rotation control (Pine Instruments). A gold disk electrode (Pine Research Instrument, 0.196 cm^2^), polished to a mirror finish (0.05 μm, Alumina), was used as the substrate. The catalyst layer was prepared by pipetting 9.88 μL of a water:ethanol (80:20 v/v) suspension of the catalyst (1 mg_catalyst_ mL^–1^), without Nafion ionomer, giving a catalyst loading of 50 μg_cat_ cm^–2^_geo_. The electrode was mounted on a rotating disk electrode shaft and dried under rotation (800 rpm) at room temperature. The oxygen evolution reaction activity was determined by linear scan voltammetry (LSV) at 20 mV s^–1^ from 1.3 to 1.8 V, rotating at 2500 rpm, and calculated at 1.51 V. The accelerated durability test (ADT) consisted of two steps of 1000 scans, each at 100 mV s^–1^ from 1.3 to 1.85 V, under rotation of 2500 rpm. The electrolyte was recovered after each electrochemical step, and the solution was levelized to 100 mL using 0.1 M HClO_4_. The specific capacitance was calculated using the average capacitance obtained every 0.1 V from 0.48 to 0.84 V using the voltammograms obtained at 100 mV s^–1^. Area-normalized capacitance values were determined from the specific capacitance and BET surface area using the method previously reported.^136^ The electrochemical surface area (ECSA) was determined from the measured specific capacitance and area-normalized capacitance (see Table S3 and supporting text in the SI), and the ECSA was used to calculate the specific activity. The Ru dissolution was monitored by inductively coupled plasma mass spectrometry (ICP-MS) using an Agilent 8900 ICP-QQQ instrument operated in MS/MS mode. A low-matrix-preset plasma condition was selected in the Agilent ICP-MS Mass Hunter software. Sample delivery was via a peristaltic pump and PFA microflow nebulizer at 0.5 mL min^–1^. The Ru standard solution was obtained from Agilent, and the stock standard solutions were diluted with the same electrolyte, 0.1 M HClO_4_, in the range of 0 to 1000 ppb. The stability number (S-number) was calculated according to previously reported methods^137,138^ and defined as the number of moles of oxygen evolved (considering 100% Faradaic efficiency) divided by the number of moles of Ru dissolved. The S-number for RuO_2_ and Ru0_.8_Ti_0.2_O_2_ was determined at three different testing stages: “initial” denotes the data before cycling; “1000” denotes the data after “initial” to 1000 cycles, and “2000” denotes the data from 1001 to 2000 cycles. The moles of Ru dissolved were determined by ICP-MS analysis of the collected electrolyte solution for each stage of the electrochemical testing. The cumulative S-number was calculated from the total current and total Ru dissolved up to 2000 cycles.

#### 2.2.3 Electron Microscopy and Spectroscopy

Annular dark-field scanning transmission electron microscopy (ADF-STEM) was performed using a JEOL NEOARM equipped with a probe corrector for STEM operated at an accelerating voltage of 200 kV and an average beam current of 10 μA. A dwell time of 8 μs was used for imaging. Electron energy loss spectroscopy (EELS) was conducted in the JEOL NEOARM equipped with a Gatan EEL spectrometer (GIF Quantum 965) and a Quantum direct electron MerlinEELS detector at 200 kV with a beam current of 10 μA. Spectra were obtained at a dispersion of 0.25 eV/channel with a dwell time of 25 ms. Background subtraction was performed in FIJI with Cornell Spectrum Imager.^1^ Principal Component Analysis (PCA) was performed in the Hyperspy UI to reduce noise. An example of typical PCA data processing is shown in Figure S1, and details are provided in the supporting text.

## 3 Results and Discussion

### 3.1 Computational Analysis of Metal Dissolution from Ru_1–*x*_Ti_*x*_O_2_-(110)

The computational surface models analyzed in this work have been previously introduced,^29,68^ and they are shown in Figure S2. For instance, each pentacoordinated (5C) metal (both Ru and Ti) is initially bonded to a subsurface oxygen right below it and to four 3-coordinated oxygen atoms (O_t_) on the surface. When fully oxidized, the 5C metal keeps oxygen coordinatively unsaturated site (O_CUS_) adsorbed to it. The hexa-coordinated (6C) metals are coordinated with two subsurface oxygen atoms, two surface O_t_ atoms, and two oxygen bridges (O_B_ atoms). After optimization of the water monolayer described in Section 2.1, c-AIMD simulations were carried out using these surfaces to evaluate the different metal dissolution processes. It is important to note that no unique dissolution mechanism has been evaluated. Instead, the analysis uses a dynamic approach in which the dissolving metal atoms are constrained by a driving force that pushes them out of the surface and into the electrolyte. The free energy is calculated and reported in the dissolution-free energy profiles in this process. No constraint is added regarding which interactions the dissolving atom must meet; therefore, for each case, the dissolution process exhibits peculiar mechanisms, as revealed by the presence of different intermediate species.

**Figure 1:**
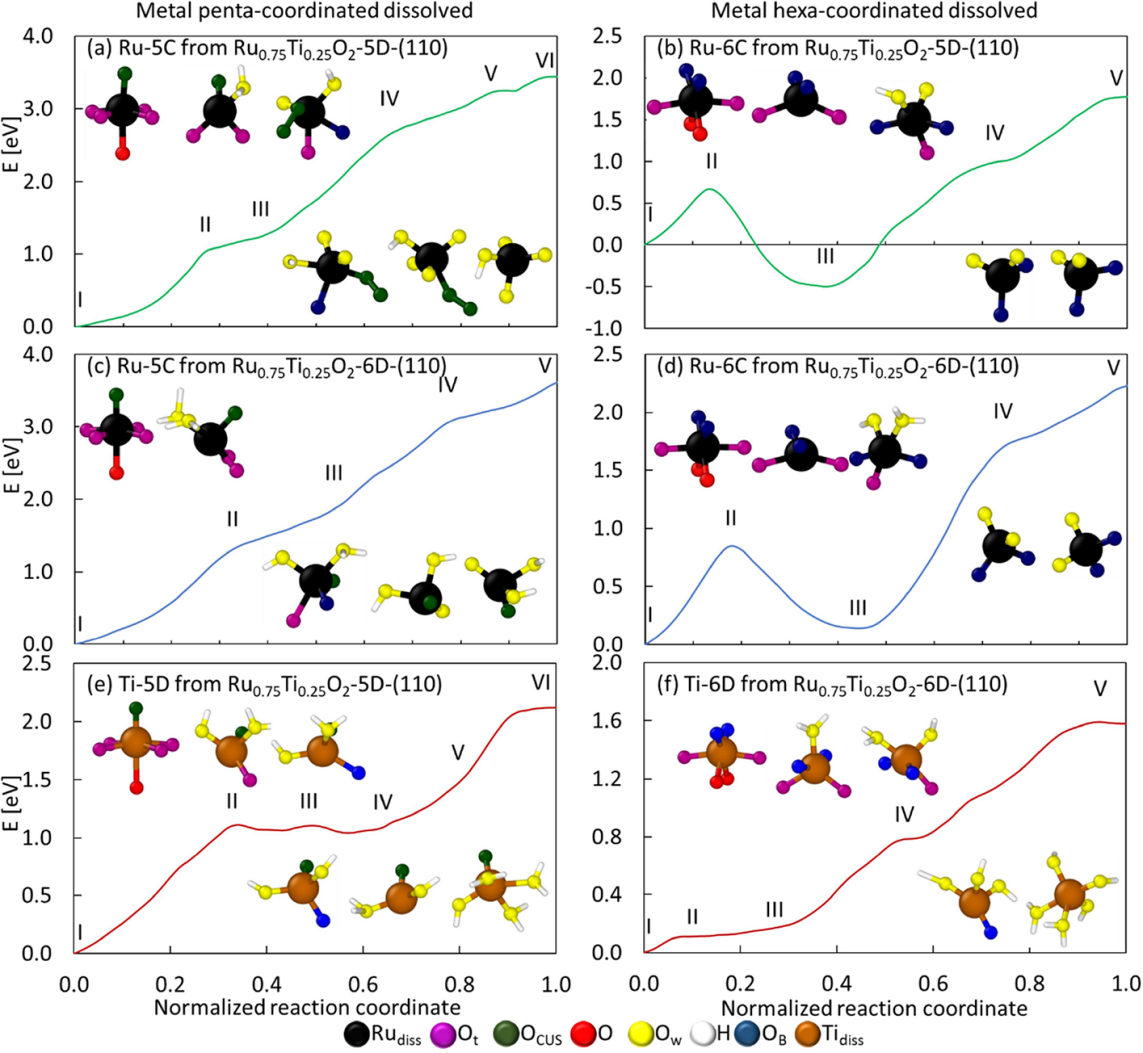
Free energy profile of ruthenium penta- (5C, first column) and hexa-coordinated (6C, second column) dissolution from the (a,b) Ru_0.75_Ti_0.25_O_2_-5D-(110), colored green; (c,d) Ru_0.75_Ti_0.25_O_2_-6D-(110) slabs, colored blue; and titanium (e) penta- (5D) and (f) hexa-coordinated (6D) from the Ru_0.75_Ti_0.25_O_2_-5D-(110) and Ru_0.75_Ti_0.25_O_2_-6D-(110) slabs, colored red, respectively. The molecular structures are representations of each estimated state. Color code in Figure. The events along each trajectory are detailed in Tables 1 and 2.

Ruthenium dissolution from the pristine RuO_2_-(110) surface was addressed in our previous work,^68^ and the free energy profiles for the Ru-5C and 6D dissolution are shown in Figure S3, along with the intermediate steps conforming to each specific dissolution mechanism. Regarding the Ru_0.75_Ti_0.25_O_2_ surfaces, Figure 1 summarizes the dissolution free energy profile of Ru penta- (5C) and Ru hexa-coordinated (6C) from surfaces substituted with (a,b) Ti penta-coordinated (5D) or (c,d) hexa-coordinated (6D), and the dissolution free energy of Ti from (e) Ru_0.75_Ti_0.25_O_2_-5D-(110) and (f) Ru_0.75_Ti_0.25_O_2_-6D-(110). The numbered states along the free energy profiles represent the evolution from the initial surface configuration to the fully dissolved compound, and the molecular structures included along each profile illustrate the initial, intermediate, and final dissolution states. More details on the atomic surface reconstructions and interfacial events during the dissolution are given in Figures S4–S6. Ru dissolution from the pristine slab was previously reported.^29,68^

Ru-5C dissolution from the Ru_0.75_Ti_0.25_O_2_-5D-(110) slab (Figure 1a) exhibits a complete uphill energy profile compared to the one from the pristine surface.^29^ Uphill energy of ∼1.08 eV is needed at state I to break three main Ru–O bonds: one with the subsurface oxygen and two of its O_t_ surface bonds, adsorbing one free water molecule before reaching state II. Compared to the dissolving state II from the pristine oxide (not shown), they share the same configuration but at ∼1 eV lower cost. With an extra energy of ∼0.24 eV, the system reaches state III, in which the dissolving metal adsorbs a second water molecule that has undergone almost complete oxidation and keeps only one of its original Ru–O_t_ bonds.

Figure S4a shows that the dissolving compound forms two new bond types with the surface. At one end, the Ru atom bonds to a neighboring O_B_ atom (expected to attract protons involved in the water oxidation steps). At the other end, an O–O bond is formed when the original O_CUS_ is attached to the dissolving Ru bonds with a second O_CUS_ adsorbed on a neighboring Ti-5D site (Figure S4a). Adding ∼1.40 eV takes the system to state IV, where all O_t_ bonds are broken, and the Ru–O_B_ bond is about to be broken. As this happens, the Ru atom adsorbs a third water molecule, while the initial two water molecules fully oxidize. Regarding the O_CUS_-O_CUS_ bond, the attraction from the dissolving compound is stronger than the attraction from the Ti-5D site, and the second O_CUS_ is desorbed from it, forming an O_2_* species adsorbed to the dissolving Ru. After the last bond with surface atoms is broken, the dissolved compound undergoes different states to become stable in aqueous media. So, from state IV to state V, it requires ∼0.53 eV to adsorb and partially oxidize a fourth water molecule, temporarily forming a RuO_6_H dissolved species (including the O_2_* species). This intermediate complex comprises a metal atom bonded to three O atoms, one OH group, and one O_2_* group. Finally, an additional ∼0.20 eV fully detached the O_2_* group to form O_2(g)_ and a fully dissolved RuO_4_H. This process shows that some of the O_CUS_ on the Ti-5D sites actively help the oxidation processes by attraction and partial adsorption of protons. This sequence is probably one of the first direct observations of O_2_ formation and evolution, suggesting that the presence of Ti may allow for spontaneous O_2_ evolution.

Ru-6C dissolution from the Ru_0.75_Ti_0.25_O_2_-5D-(110) slab (Figure 1b) shows an interesting free energy profile, comparable to the corresponding on the pristine surface.^68^ For instance, both show stabilization in state II, denoted by negative free energy, signaling the system’s preference to keep in such a state. The initial energy cost from state I to II is ∼0.67 eV, compared to ∼0.48 eV for the RuO_2_-(110) pristine surface for the dissolving Ru breaking two bonds with the subsurface O atoms. Next, the system is stabilized by a series of molecular events: adsorption and full oxidation of a first water molecule, adsorption and partial oxidation of a second water molecule, and one Ru–O_t_ bond breaking; all this while reaching state III, in a downhill pathway of ∼−1.17 eV from state II. At this state, some of the O_CUS_ on Ti-5D help in the oxidation steps revealed by the formation of HO* groups on those sites. However, to undergo dissolution, the dissolving system requires ∼1.50 eV to reach state IV, where the two adsorbed water molecules are fully oxidized. Then, the dissolving metal attracts and detaches an O_B_ atom from the surface, creating a new type of Ru-5C, just as that in the original RuO2-(110) surface. Next, it attracts and detaches another OB, forming a second Ru-5C of this new type. Finally, the dissolved RuO_4_ compound incorporates and stabilizes into the aqueous media at an energy expense of ∼0.76 eV until it reaches the final state V. Detailed molecular descriptions for each state are given in Figure S4b. In summary, in these surfaces where Ti is located in a 5C site, the Ru-5C dissolution event triggers the spontaneous O2 formation, and the Ru 6C dissolution promotes the formation of new active sites of the Ru-5C type, in both cases favorable to OER catalysis.

The dissolution of Ru-5C from the Ru_0.75_Ti_0.25_O_2_-6D-(110) slab shown in Figure 1c is comparable to the dissolution of the same metal from the 5D surface (Figure 1a), suggesting that the Ti atom location does not primarily affect the Ru dissolution process. Table 1 describes the events and energies at each step during energy profile evolution. Detailed molecular descriptions are given in Figure S5a. Moreover, the Ru-6C dissolution from the Ru_0.75_Ti_0.25_O_2_-6D-(110) slab free energy profile (Figure 1d) is similar to the one from the Ru_0.75_Ti_0.25_O_2_-5D-(110) slab (Figure 1b). However, steps II to III do not show negative free energy like the 5D surface, which suggests less surface stabilization. Table 1 lists the individual events. The detailed molecular description is given in Figure S5b.

**Table 1:** Dissolution of Ru-5C and Ru-6C from Ru_0.75_Ti_0.25_O_2_ Surfaces

energy barrier (eV)	event	physical processes for dissolution of Ru-5C from Ru_0.75_Ti_0.25_O_2_-6D-(110)
1.08	I → II	Ru-subsurface O bond broken; 2 Ru–O_t_ bonds broken; 1st H_2_O molecule adsorbed
0.24	II → III	Ru–O_t_ bond broken; 1st adsorbed H_2_O fully oxidized; 2nd H_2_O is adsorbed and partially oxidized; Ru–O_B_ bond formed; O_cus-_O_cus_ bond formed.
1.40	III → IV	dissolving complex separates from surface; 2nd H_2_O fully oxidized; 3rd H_2_O adsorbed and partially oxidized; O_2_* attached to dissolving Ru
0.53	IV → V	3rd H_2_O fully oxidized; 4th H_2_O adsorbed and partially oxidized; RuO_6_H dissolved species (including the O_2_* species)
0.20	V → IV	O_2_* detaches from dissolving Ru; RuO_4_H stable dissolved species

energy barrier (eV)	event	physical processes for dissolution of Ru-6C from Ru_0.75_Ti_0.25_O_2_-5D-(110)
0.67	I → II	subsurface bonds broken
	II → III	downhill pathway; Ru–O_t_ bond broken; 2 H_2_O adsorption and oxidation
1.50	III → IV	one Ru–O_t_ bond broken; one O_B_ detached from surface creating new Ti 5C-type site.
0.76	IV → V	HO* is oxidized; second O_B_ detached from surface and the RuO_4_ dissolved compound is stabilized in the aqueous medium

Next, we followed Ti dissolution. Ti-5D dissolution (Figure 1e) shows a lower free energy profile compared to the ones from Ru-5C on the same surfaces. Microscopic events and energy barriers are listed in Table 2, and a detailed molecular description is given in Figure S6a. Ti-6D dissolution exhibits the overall lowest free energy profile (Figure 1f). Interestingly, initial dissolution is very favorable, and most of the energy cost is associated with the solvation energy of the dissolved compound incorporated into the bulk electrolyte. Detailed molecular description is given in Figure S6b.

**Table 2:** Dissolution of Ti-5D and Ti-6D Atoms

energy barrier (eV)	event	physical processes for dissolution of Ti-5D from Ru_0.75_Ti_0.25_O_2_-5D-(110)
1.10	I → II	3 Ti–O_t_ surface bonds broken; Ti–O subsurface bond broken; 2 H_2_O molecules adsorbed, one partially oxidized
	II → III	last Ti–O_t_ bond broken; Ti–O_B_ formed.
	III → IV	water oxidized; 2 HO* adsorbed on dissolving species.
0.31	IV → V	Ti–O_B_ broken; TiO(OH)_2_ dissolved
0.64	V → VI	TiO(OH)(H_2_O)_3_ final dissolved species

energy barrier (eV)	event	physical processes for dissolution of Ti-6D from Ru_0.75_Ti_0.25_O_2_-6D-(110)
0.10	I → II	Ti breaks bonds with 2 subsurface O atoms; 1 H_2_O molecule adsorbed (acts as Ti 5C active site)
0.17	II → III	Ti–O_t_ bond broken; 2 H_2_O adsorbed and one oxidized
0.53	III → IV	last Ti–O_t_ bond broken; second water partially oxidized; 3rd water adsorbs
0.79	IV → V	dissolved compound Ti(OH)_3_(H_2_O)_2_ is stabilized in the aqueous medium

As reported in previous dissolution analyses, RuO_4_ seems to be the stable dissolved species from Ru dissolution regardless of its initial surface configuration, Ru-5C or 6C.^9^ Even though the close solvation shell keeps the Ru-5C dissolution product from the Ru_0.75_Ti_0.25_O_2_-5D- and 6D(110) slab as RuO_4_H and RuO_4_H_2_ species, respectively, we hypothesize that at longer simulation times, the HO* groups will be oxidized to finally form the RuO_4_ species. Moreover, Manoharan and Goodenough demonstrated that the deprotonation potential of RuOH-like species on RuTi and mixed-metal oxide surfaces may induce a shift in the corrosion reaction toward more anodic potentials without a pronounced variance in the potential at which oxidation reactions take place.^139^ This underscores a nuanced interplay with significant implications for the overall stability of the metal dissolution processes. Furthermore, the dissolution of Ti also gives the same species regardless of its initial configuration, with a final dissolved product in the form of TiO_5_H_7_, which seems to be stable up to the reached simulation time. The reported Ti(OH)_3_(H_2_O)_2_ and TiOOH(H_2_O)_3_ dissolved structures were stable within the simulation timeframes, which were enough to evaluate the dissolution of the dissolving compound from the surface, followed by its stable incorporation into the aqueous media. After dissolution was reached and no interaction with the surface was observed, the simulation was ended after the appearance of a species that incorporated into the electrolyte can stabilize the free energy, which reaches a plateau as a function of the reaction coordinate. In the case of the dissolved Ti-species, a hydrogen bond solvation is found for most of the H in the compounds with the closest electrolyte water molecules, while a few other H atoms coordinate with O_CUS_ atoms. It is interesting that the dissolved species have a Ti oxidation state of +3. Recent work observed similar oxidation/dissolution behavior in Ni dissolution from LiNiO_2_,^140^ where after surface protonation, Ni (normally in +3 state) is reduced to Ni^2+^ which is easily dissolved due to weakened interactions with surface O atoms. In this work, the reported Ti(OH)_3_(H_2_O)_2_ and TiOOH(H_2_O)_3_ structures may be stabilized by H-bond interactions. Moreover, Figure S7 shows the detailed interaction between the Ti-dissolving compounds and the solvation shell from the surrounding media. There is a strong hydrogen bond network that stabilizes the Ti(OH)_3_(H_2_O)_2_ and TiOOH(H_2_O)_3_ structures that take place along with the electron density redistribution, as shown in the isosurfaces in the regions of electron density accumulation (yellow) and depletion (blue) in Figure S7. More details on the electron density distribution are given next.

**Figure 2:**
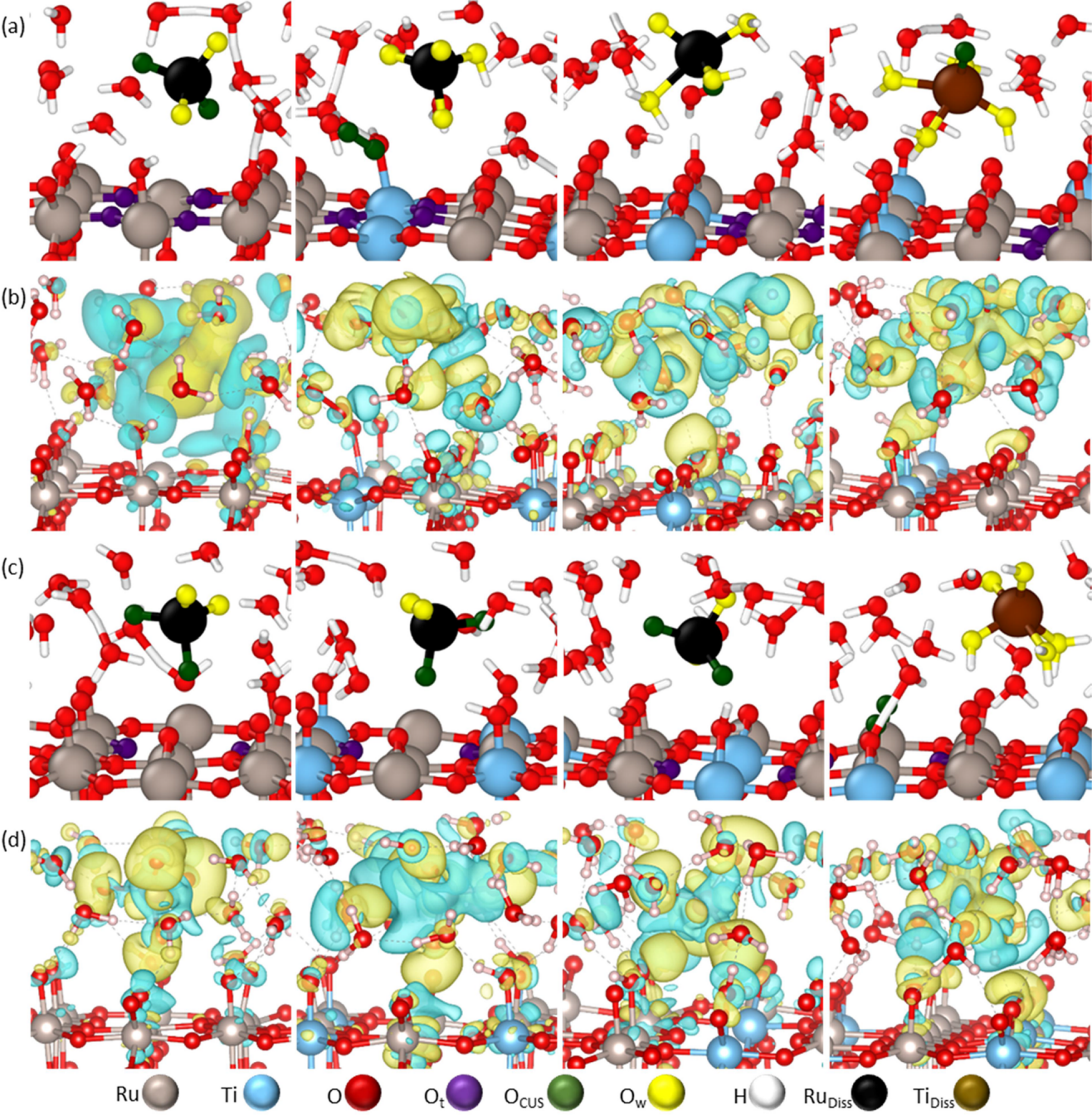
Final dissolution product reported in the free energy profiles for (a) Ru-5C dissolution from the pristine RuO_2_-(110), Ru_0.75_Ti_0.25_O_2_-5D-(110), and Ru_0.75_Ti_0.25_O_2_-6D-(110) slabs, and Ti-5D dissolution from the Ru_0.75_Ti_0.25_O_2_-5D-(110) slab, respectively; (b) electron density distribution for the final dissolution products shown in (a); (c) final dissolution product reported in the free energy profiles for Ru-6C dissolution from the pristine RuO_2_-(110), Ru_0.75_Ti_0.25_O_2_-5D-(110), and Ru_0.75_Ti_0.25_O_2_-6D-(110) slabs, and Ti-6D dissolution from the Ru_0.75_Ti_0.25_O_2_-6D-(110) slab, respectively; and (d) electron density distribution for the final dissolution products shown in (c). Electron density accumulation: yellow; electron density depletion: blue.

When the final state of dissolution is achieved, we analyze the effects of electron redistribution in the interfacial region, comparing Ru and Ti dissolution from the pristine and Ti-substituted surfaces (Figure 2). In contrast to Figures S4–S6 and 2a,c illustrate most of the water molecules involved in the dissolution process, revealing their interaction with the dissolved compound. Figure 2b,d reveal contrasting effects due to Ti’s presence and the different natures of the dissolving compounds. Each image has two clear interfacial regions: the “electrolyte side” and the “surface side.” The pristine surface is highly localized around the dissolved complex (Figure 2b, left), whereas the surface shows some small electron-depleted regions (blue) after Ru-5C dissolution. On the Ti-substituted cases (two middle Figure 2b images), some electron delocalization is observed around the dissolving complex and electron accumulation on the surface (yellow), especially on oxygen atoms with O_B_ and O_t_ configuration both with Ru-5C and with Ti dissolution (right side Figure 2b image). Note that electron delocalization on the dissolved complex denotes changes in its chemistry (the presence of OH groups) and stabilization in solution. In contrast, the electron changes on the surface due to coordination imbalances caused by Ru dissolution directly affect the catalytic reactions. These effects on the electronic density distribution trigger different performances on surface activity regarding water-splitting steps compared to those of the activity prior to dissolution. The results of the activity after dissolution calculations are presented later in this work.

The computational analysis of metal dissolution at a higher Ti concentration (50% Ti) on the cleaved Ru_0.50_Ti_0.50_O_2_-(110) surface reveals key insights into dissolution mechanisms for both Ru and Ti and the detailed analysis of these results is given in Supporting Information, under “Computational Analysis of Metal Dissolution at a Higher Concentration of the Substituent: 50% Ti”. In summary, Ru dissolution was found to be highly unfavorable due to a high energy cost, forming a RuO_4_* cluster on the Ti-5D active sites. Ti-5D dissolution, although requiring less energy than Ru, indicates potential Ru enrichment upon further electrochemical activity. Ti-6D dissolution displayed two distinct energy regions, with the spontaneous involvement of neighboring Ti-6D atoms, which attracted and adsorbed water molecules, indicating structural instability at a 50% Ti concentration. This leads to phase separation and metal aggregation, suggesting that while Ru is stabilized initially, the overall system becomes unstable, prompting further Ti dissolution to reach a more stable configuration at a lower Ti content. Additionally, the frequent formation of hydroperoxide species in the presence of Ti due to electrolyte oxidation was observed, further impacting the system stability.

### 3.2 Surface Reconstruction after Metal Dissolution

**Figure 3:**
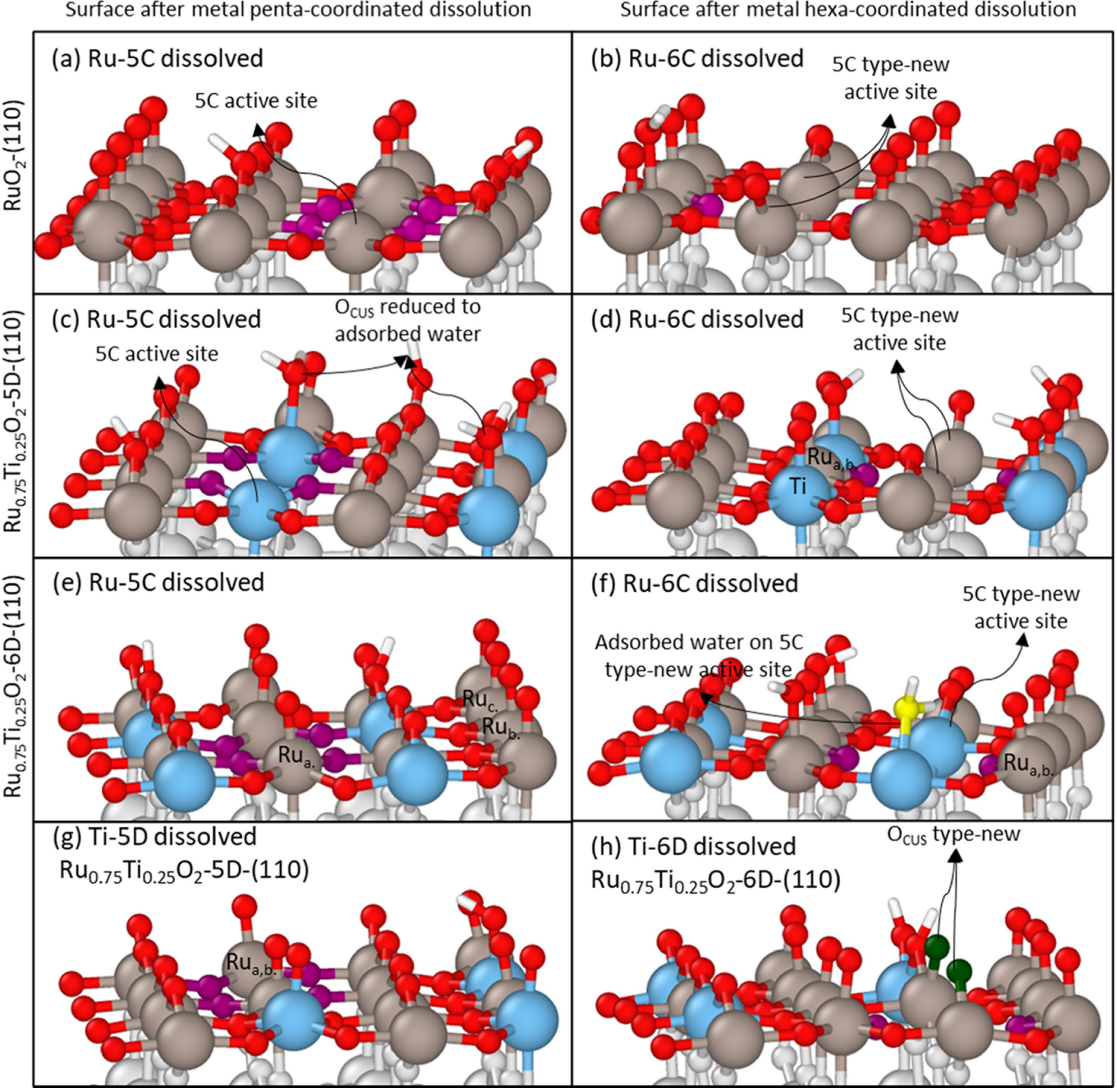
Surface configurations after ruthenium penta- (left column) and hexa-coordinated (right column) dissolution from (a,b) RuO_2_-(110), (c,d) Ru_0.75_Ti_0.25_O_2_-5D-(110), (e,f) Ru_0.75_Ti_0.25_O_2_-6D-(110); surface after titanium dissolution for (g) Ti-5D dissolution from Ru_0.75_Ti_0.25_O_2_-5D-(110) and (h) Ti-6D dissolution from Ru_0.75_Ti_0.25_O_2_-6D-(110). Silver: Ru, blue: Ti; red: O, white: H; purple: Ot bonded to the dissolving atom; green: O_B_ bonded initially to the dissolving atom; yellow: O from the free water molecule.

As described above and in more detail in the Supporting Information, the surface with a 50% concentration of Ti does not show structural stability. Instead, the dynamic evolution suggests that an excess of Ti tends to leave the surface until it reaches a lower, more stable concentration. To investigate the stability of lower Ti surface concentrations, we evaluated the surface reconstruction and activity after dissolution from the Ru_0.75_Ti_0.25_O_2_-5D and 6D-(110) slabs. Surface configurations after dissolution are listed in Figure 3. During dissolution, several water molecules become oxidized by the dissolving metal or by the surface-active oxygen atoms, both O_CUS_ and O_B_ (Figure 3). Ru-5C dissolution from the RuO_2_-(110) surface (Figure 3a) shows the formation of penta-coordinated active sites after the dissolving metal attracts their adsorbed O_CUS_. Ru-6C dissolution (Figure 3b) induces the formation of new Ru-5C active sites when the dissolving Ru-6C pulls its O_B_ atoms to form a part of the dissolving compound. On the Ti-substituted surfaces, some of the O_CUS_ on top of Ti-5D sites tend to attract H atoms from the oxidized water molecules that actively participate in the dissolution process and form HO* or a few H_2_O* adsorbed species. However, the O_CUS_ atoms on top of Ti-5D sites show good stability as, in most cases, the O_CUS_ sites remain after the dissolution and the associated proton exchange processes. Compared to that of O_CUS_ on top of Ru, higher activity is registered on the O_B_ bonded to the surface Ti-6D atoms, attracting H atoms and helping the oxidation processes. The Ru–O bond is considerably stronger than the Ti–O bond, which leads to Ti-5D new active sites after the dissolution of Ru-5C (Figure 3c). Similar new active sites arise when Ru-6C dissolves, taking away the attached O_B_ and leading to the formation of new penta-coordinate active sites after dissolution (Figure 3d,f), and new O_CUS_ sites are found after Ti-6D dissolution (Figure 3h). In contrast, less reconstruction is observed in Figure 3e for the 6D substituted surface.

It is worth noting that surface atoms from the reported Ru_0.75_Ti_0.25_O_2_-5D and 6D-(110) slabs do not exhibit significant rearrangements, and the main (110) features are preserved. These structural preservation features are in addition to the formed vacancies discussed above. The behavior of these surfaces shows significant differences concerning the Ru_0.50_Ti_0.50_O_2_ surface rearrangement shown in Figures S9 and S10 and described in the dissolution section and to the higher rearrangement on the Zr-substituted surfaces reported in our previous work.^68^

### 3.3 Metal Dissolution Effects on the Surface Electronic Structure

Density of states (DOS) calculations were carried out for specific atoms (on the RuO_2_-(110) and the Ti-5D and 6D substituted surfaces). The analysis included the Ru-4d-band and the O-2p-band center shifts relative to the surface before dissolution, and the DOS was evaluated for Ru-5C, O_B_, and O_CUS_ atoms closest to the surface vacancy left by the dissolved atom. The d-band center shift is defined as [d-band center—d band center (before dissolution)] in eV. A positive shift indicates a d-band center shift toward the Fermi level, suggesting higher reactivity. Similarly, the 2p-band center shift is defined as [2p-band center- 2p band center (before dissolution)] in the eV of the O_B_ atom closest to the dissolving atom. A positive shift indicates a 2p-band center shift toward the Fermi level, suggesting higher reactivity. Table 3 shows that Ru-5C dissolution has a favorable effect on the 4d-band center of the Ru 5C atoms closest to the surface vacancy on the pristine and 5D substituted surfaces, shifting the d-band center closer to the Fermi level (less negative energies), thus suggesting a more active surface. However, the shift has the opposite direction on the 6D surface. On the other hand, the dissolution of Ru-6C shows a shift to more negative binding energy only on the 5D surface, whereas the change suggests more active Ru-5C atoms on the pristine and 6D surfaces. The highest 4d-band center shifts toward more active surfaces, shown in Table 3, belong to the Ru-5C-type new sites that appear after Ru-6C dissolution in both the pristine RuO_2_-(110) and the Ru_0.75_Ti_0.25_O_2_-5D surfaces, respectively.

**Table 3:** Ru-4d-Band Center Shift (eV)

dissolving atom	d-band center shift (eV) of Ru-5C atom closest to the dissolving atom			d-band center shift (eV) of new Ru-5C active site created after dissolution	
	pristine RuO_2_ surface	Ti-5D surface	Ti-6D surface	pristine RuO_2_ surface	Ti-5D surface
Ru-5C	+0.25	+0.20	–0.28	N/A	N/A
Ru-6C	0.03	–0.14	0.08	0.54	0.61
Ti-6D	N/A	N/A	0.15	N/A	N/A
Ti-5D	N/A	–0.20	N/A	N/A	N/A

Regarding the effect of metal dissolution on the O_B_ atoms closest to the surface vacancy, the most significant shift toward a lower reactivity region occurs on the RuO_2_-(110) surface after Ru-5C dissolution, reported in Table 4, with a shift of 0.9 eV or 36% with respect to the pristine surface before dissolution (−3.42 vs −2.52 eV, respectively). This behavior is explained by the surface structure reported in Figure 3, where hydrogen atoms remain attached to some of the O_B_ sites surrounding the vacancy site. However, it is essential to note that in the actual electrolysis conditions and the presence of the operative electric potential, these hydrogen atoms may detach, releasing the protons and electrons that will undergo the hydrogen evolution reaction. So, the considerably lower reactivity may be a function of the reduced OH^#^ state of the O_B_ sites instead of a site characteristic. Moreover, Ru-6C dissolution has a negligible effect on the behavior of the O_B_-2p-band center behavior. The Ru_0.75_Ti_0.25_O_2_-5D surface has a considerably low impact on the O_B_-2p-band center under all of the dissolution scenarios evaluated. Finally, Ti-6D sites on the surface induce the largest O_B_-2p-band shifts compared to the pristine RuO_2_-(110) surface atoms.

**Table 4:** O_B_-2p-Band Center Shift (eV) of the O_B_ Atom Closest to the Dissolving Atom

dissolving atom	2p-band center shift (eV) of O_B_ atom closest to the dissolving atom		
	pristine RuO_2_ surface	Ti-5D surface	Ti-6D surface
Ru-5C	–0.90	0.04	–0.11
Ru-6C	0.00	0.01	–0.05
Ti-6D	N/A	N/A	–0.65
Ti-5D	N/A	0.09	N/A

Finally, the 2p-band center was also evaluated in the O_CUS_ atoms as they are expected to participate to some degree in the different OER mechanisms. This is shown by the dynamic simulations where they participate actively in deprotonation steps and, in specific cases, in the direct oxygen recombination mechanisms. These results are reported in Table 5. The pristine RuO_2_-(110) surface shows the lowest O_CUS_-2p-band center of all systems evaluated, which is in line with the AIMD simulations reported previously, where the presence of Ti on the surface triggered the participation of surface oxygen atoms in spontaneous deprotonation reactions.^29^ After Ru-5C and Ru-6C dissolution, the O_CUS_ atoms suffer an O 2p-band center shift toward higher energy values than the surface before dissolution (−2.11 and −2.44 eV, respectively, vs −2.81 eV). Moreover, a similar trend occurs for metal dissolution from the Ru_0.75_Ti_0.25_O_2_-5D and 6D surfaces. However, in the 5D surface, dissolution of Ru-6C decreases the O_CUS_-2p-band center toward a lower value of −2.70 eV compared to the −2.42 eV from the same surface before dissolution, signaling lower reactivity.

**Table 5:** 2p-Band Center Shift (eV) of O_CUS_ Atom Closest to the Dissolving Atom

dissolving atom	2p-band center shift (eV) of O_CUS_ atom closest to the dissolving atom		
	pristine RuO_2_ surface	Ti-5D surface	Ti-6D surface
Ru-5C	+0.70	0.17	0.16
Ru-6C	0.37	–0.28	0.07
Ti-6D	N/A	N/A	0.03
Ti-5D	N/A	0.29	N/A

These results revealed significant changes in the reactivity indicators after dissolution, such as the Ru-4d and O-2p electronic band centers of Ru and O atoms located near surface vacancies upon metal dissolution. Specifically, Ru-5C dissolution affected the Ru-5C 4d-band center, resulting in a noteworthy shift toward lower binding energies, signaling increased reactivity. At the same time, a similar, but less marked, trend is observed for the dissolution of Ru-6C. However, new catalytically active Ru-5C-type sites emerged after Ru-6C dissolution in both pristine RuO_2_ and Ru_0.75_Ti_0.25_O_2_-5D surfaces, significantly increasing the activity index. Notably, a divergent trend was observed between Ru_0.75_Ti_0.25_O_2_-5D and 6D surfaces, with Ru-5C dissolution decreasing the 4d-band center in the former but increasing it in the latter. Evaluation of the O-2p-band centers revealed varied effects on oxygen atoms adjacent to vacancies. The dissolution of Ru-5C led to a substantial shift toward lower reactivity. However, hydrogen atoms at some oxygen sites may influence this behavior. In contrast, Ru-6C dissolution shows no significant effect on the center of the O-2p-band center. For the Ru_0.75_Ti_0.25_O_2_-5D surface, minimal shifts occurred after various dissolution scenarios, mostly toward lower binding energies. Ti-6D dissolution induced the most significant O-2p-band shifts, particularly toward higher reactivity regions.

### 3.4 Computational Analysis of OER Energy Barriers after Metal Dissolution

Oxygen evolution activity was evaluated following the surface configurations resulting after dissolution (Figure 3), identifying the nonequivalent active sites, and considering the aqueous media effect on oxidation steps. Some active sites in Figure 3 are labeled as (a) and (b), indicating that the same active site was used to evaluate different oxidation pathways: one regarding surface site participation (a) and another regarding oxidation via electrolyte attraction of detached protons (b). A third site labeled as c. in Figure 3e shows the effect of the active site location relative to the vacancy site formed compared to a and b. sites. Following these changes in the atomic and electronic structure induced by metal dissolution, we evaluated their effect on the free water-oxygen association OER mechanism energetic barriers. Although in previous reports, we considered a complex reaction network with multiple possible mechanistic pathways, the direct oxygen and oxygen-hydroxyl recombination mechanisms were found to be less probable to occur due to their considerably higher activation energies.^29,68^ Here, the analysis is done over the most likely steps based on the previous and current observations for the free water-oxygen association mechanism^141^:

1. free water molecule–oxygen association step, H_2_O_(l)_ + O* → H^#^ + *OOH
2. hydroperoxyl oxidation step, *OOH → *OO + H^#^, and
3. oxygen release helped by water adsorption, *OO + H_2_O_(l)_ → O_2(g)_ + H_2_O* (not included in the previous analyses).

Table 6 summarizes the activation energy for each reaction step and shows in bold the low activation energies that define possible reaction pathways for each surface. The first column in Table 6 shows each of the surfaces evaluated, starting from the pristine surfaces (before dissolution) and the surface after metal dissolution. Starting with the pristine RuO_2_-(110) surface, it shows an activation energy of 1.64 eV for the free water molecule–oxygen association step. After metal dissolution occurs, the effect on the activation energy on the surface remaining after dissolution highly depends on the original configuration of the dissolving metal; hence, after Ru-5C dissolves, the activation energy goes up to 1.76 eV while after Ru-6C dissolution, it decreases to 0.71 eV. When evaluating the hydroperoxyl oxidation step activation energy, the pristine surface exhibits a considerably low value of 0.26 eV. In comparison, on the surface after dissolution, it goes up 3-fold (0.76 eV) and 4-fold (1.08 eV) after Ru-5C and Ru-6C dissolution, respectively. Finally, the activation energy for the oxygen release helped by the water adsorption step shows similar values regardless of the dissolution state on the surface: 1.54, 1.57, and 1.62 eV on the pristine and after Ru-5C and Ru-6C dissolution, respectively. These different ranges of activation energies from the pristine RuO_2_-(110) surface show that metal dissolution alters the reaction mechanism, shifting the rate-determining step (rds) according to the rate of Ru-5C to Ru-6C dissolved.

**Table 6:** Activation Energy for the Free Water Molecule–Oxygen Association Step (H_2_O_(l)_ + O* → H^#^ + *OOH); Hydroperoxyl Oxidation Step (*OOH → *OO + H^#^); and Oxygen Release by Water Adsorption (*OO + H_2_O_(l)_ → O_2(g)_ + H_2_O*) for Pristine and after Dissolution Surfaces^a^

surface		ads. site	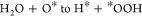	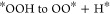	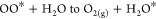
			*E*_a_ (eV)	*E*_a_ (eV)	*E*_a_ (eV)
RuO_2_	pristine	Ru	1.64	0.26	1.54
	Ru-5C_dis_	Ru	1.76	0.76	1.57
	Ru-6C_dis_	Ru	0.71	1.08	1.62
Ti-5D	pristine	Ru	1.89	0.00	1.29
		Ti	2.10	0.00	0.00
	Ru-5C_dis_	Ru	2.07	0.00	1.24
		Ti	1.01	0.00	0.95
	Ru-6C_dis_	Ti	1.89	1.42	0.90
		Ru_a_	2.02	0.60	1.19
		**Ru**_**b**_	**1.38**	**0.00**	**1.19**
	Ti-5D_dis_	**Ru**_**a**_	**1.01**	**0.00**	**1.16**
		**Ru**_**b**_	**1.37**	**0.68**	**1.16**
Ti-6D	pristine	Ru	1.48	0.00	1.43
	Ru-5C_dis_	Ru_a_	1.91	0.83	1.37
		Ru_b_	1.72	0.83	1.37
		**Ru**_**c**_	**1.47**	**0.00**	**1.37**
	Ru-6C_dis_	Ru_a_	1.75	0.89	1.59
		Ru_b_	2.13	0.00	1.59
	Ti-6D_dis_	Ru	2.09	0.96	1.48

a Subindexes a and b indicate that the same active site was used to evaluate different oxidation pathways: one regarding surface site participation (a) and another regarding oxidation via electrolyte attraction of detached protons (b). Subindex c in the Ti-6D surface shows the effect of the active site location relative to the vacancy site formed compared to a and b sites

Regarding the effect of Ti substitution on surface activity before and after dissolution, the Ti-5D substitution on the Ru_0.75_Ti_0.25_O_2_-5D-(110) surface exhibits a higher activation energy for the free water molecule–oxygen association step: 1.89 and 2.10 eV on the Ru and Ti active sites, respectively. After Ru-5C dissolution, this does not improve for the remaining Ru active sites on the surface, where the activation energy goes up to 2.07 eV. Even though there is a considerable improvement in the Ti active site, where the activation energy for the first step (free water molecule–oxygen association) goes down to 1.01 eV, and this step remains as the rds, the results discussed above regarding the faster Ti dissolution compromises the actual stability of these sites and their contribution to the overall improved activity. However, when Ru-6D and Ti-5D dissolution occurs, activity improvement is observed: after Ru-6D dissolved, the Ru_b_ reaction pathway evaluated (oxidation via electrolyte attraction of detached protons) shows 1.38 eV activation energy for the free water molecule–oxygen association step, lower compared to the pristine RuO_2_-(110) and Ru_0.75_Ti_0.25_O_2_-5D-(110) surfaces, and to the RuO_2_-(110) after metal dissolution. Moreover, after Ti-5D dissolution, the remaining Ru active sites exhibit lower activation energies of 1.01 and 1.37 eV, depending on the deprotonation path evaluated. However, deprotonation via surface site participation (1.01 eV activation energy for the free water molecule–oxygen association step) shows a shift of the rds to the oxygen release helped by the water adsorption step with an activation energy of 1.16 eV.

Finally, the effect of Ti-6D substitution is less relevant regarding the activity improvement on the surface before and after dissolution. However, one can observe that after Ru-5C dissolution, the active sites on the opposite side to the newly formed vacancy (Ru_c_) exhibit slightly lower activation energy for the first reaction step, which remains as the rds for these sites. These results show a complex relationship between the evaluated mechanism and the dynamic surface reconstruction after dissolution that has been demonstrated to affect the activation energies and rds evaluated.

### 3.5 Experimental Analysis of the Effect of Metal Dissolution on OER Activity and Electrochemical Stability

**Figure 4:**
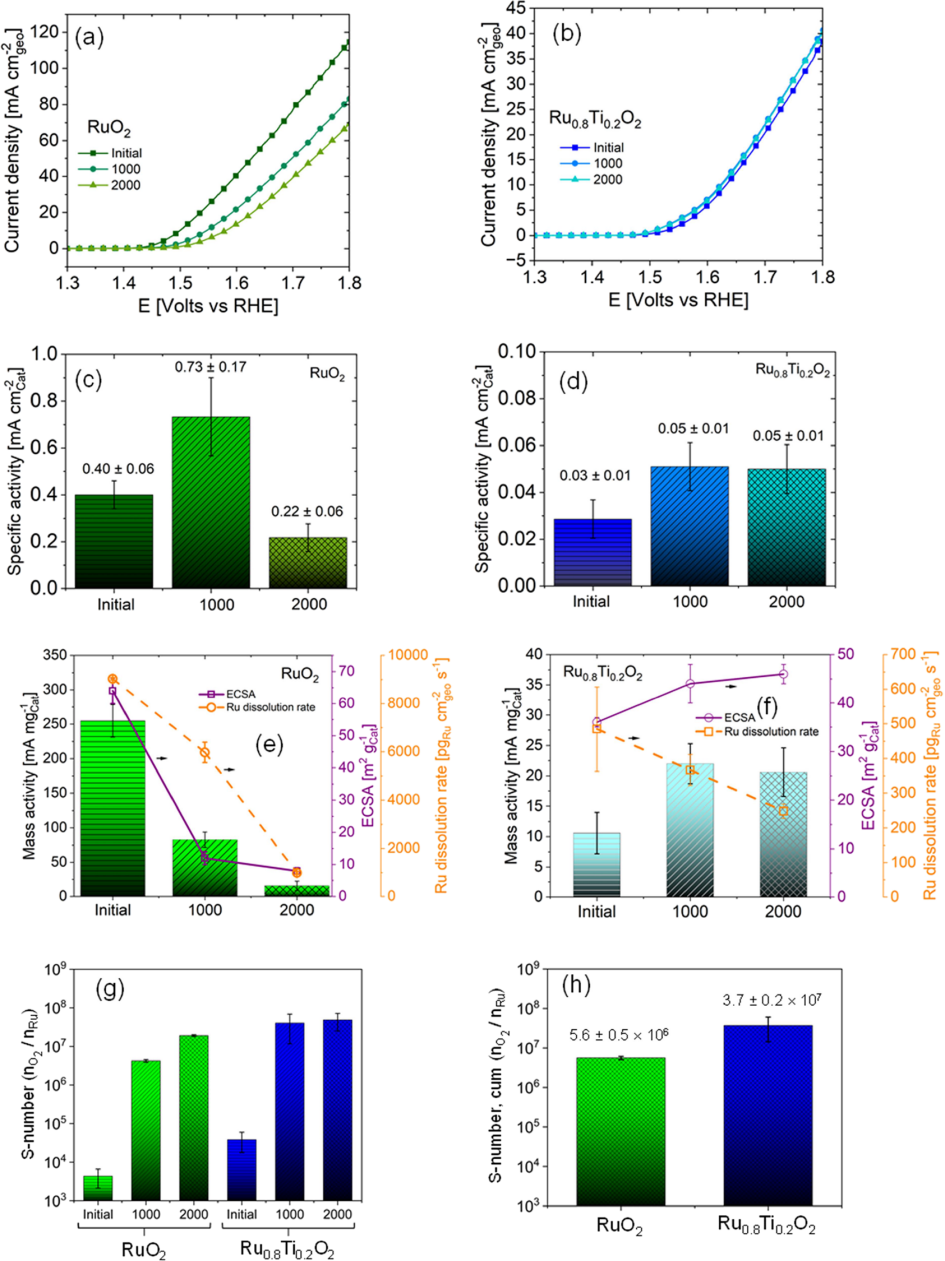
Linear scan voltammetry (at 20 mV s^–1^) in the oxygen evolution region during the electrochemical evaluation protocol of RuO_2_ (a) and Ru_0.8_Ti_0.2_O_2_ (b) using 50 μg cm^–2^ of catalyst and tested in Ar-purged 0.1 M HClO_4_ under rotation at 2500 rpm. The accelerated durability test (ADT) consisted of cycling at 100 mV s^–1^ from 1.3 to 1.85 V vs RHE in two steps of 1000 cycles; “initial” denotes the data before cycling; “1000” denotes the data after “initial” to 1000 cycles, and “2000” denotes the data from 1001 cycles to 2000 cycles. Comparison of specific activities of RuO_2_ (c) and Ru_0.8_Ti_0.2_O_2_ (d) calculated at 1.51 V. Comparison of the mass activity, electrochemical surface area (ECSA), and dissolution rate of Ru of RuO_2_ (e) and Ru_0.8_Ti_0.2_O_2_ (f). Comparison of RuO_2_ and Ru_0.8_Ti_0.2_O_2_ stability numbers (S-number) for initial, 1000 cycles, and 2000 cycles (g). Comparison of cumulative S-number (S-number, cum) for RuO_2_ and Ru_0.8_Ti_0.2_O_2_ (h).

To compare with our computational results, we experimentally evaluated RuO_2_ and Ru_0.8_Ti_0.2_O_2_ before and after a two-step durability test that involved sweeping to a high potential (1.85 V) to determine how the OER activity, surface, and metal dissolution change over the durability tests and how Ti substitution influences the electrochemical stability (Figure 4). We previously reported structural characterization of the initial RuO_2_ and Ru_0.8_Ti_0.2_O_2_ materials using X-ray diffraction, X-ray photoelectron spectroscopy, and scanning/transmission electron microscopy analysis.^29^ The linear scan voltammetry (LSV) results for RuO_2_ and Ru_0.8_Ti_0.2_O_2_ (Figure 4a,b) showed that RuO_2_ exhibited a consistent decrease in current after 1000 and 2000 cycles, while the current of Ru_0.8_Ti_0.2_O_2_ remained relatively stable and even slightly increased. We calculated the specific activity of RuO_2_ and Ru_0.8_Ti_0.2_O_2_ (Figure 4c,d) using the electrochemical surface area (details provided in Table S3 and supporting text in SI). The analysis of the OER-specific activities supports the idea that the OER surface changes with cycling, and there are significant differences between RuO_2_ and Ru_0.8_Ti_0.2_O_2_. The OER-specific activity of RuO_2_ initially increased after 1000 cycles and then decreased after 2000 cycles. In contrast, the level of the OER-specific activity of Ru_0.8_Ti_0.2_O_2_ increases after 1000 cycles and remains stable with further cycling. The experimentally observed changes in specific activity with cycling are consistent with our computational analysis that shows that the removal of Ru or Ti atoms from the surface leads to changes in the density of states (DOS), the d-band center, and activation energies for the OER reaction steps on newly formed active sites.

To further understand the relationships between changes in activity, electrochemical surface area, and metal dissolution with cycling, we plotted these values together for RuO_2_ and Ru_0.8_Ti_0.2_O_2_ (Figure 4e,f). The loss of mass activity of RuO_2_ during the first 1000 cycles occurs from a significant reduction of ECSA of 80% despite a substantial increase in the specific activity. Further cycling of RuO_2_ results in additional lowering of the mass activity and ECSA. For RuO_2_, a high Ru dissolution rate of 9033 ± 43 pg_Ru_ cm^–2^_geo_ s^–1^ is observed after 1000 cycles, which lowers to 989 ± 48 pg_Ru_ cm^–2^_geo_ s^–1^ after 2000 cycles. For the case of RuO_2_, changes in the mass activity of RuO_2_ with cycling are directly correlated with Ru dissolution and lowering of the ECSA (Figure 4e), resulting in fewer active sites available despite the initial increase in specific activity (Figure 4c). For Ru_0.8_Ti_0.2_O_2_, different behavior was observed with cycling. The OER mass activity of Ru_0.8_Ti_0.2_O_2_ increased after 1000 cycles and then remained stable (Figure 4c), which can be explained by metal dissolution, which increases the electrochemical surface area and new active sites. The Ru dissolution from Ru_0.8_Ti_0.2_O_2_ after 1000 cycles (484 ± 122 pg_Ru_ cm^–2^_geo_ s^–1^) is 19 times lower than that for RuO_2_ and further decreased to 248 ± 20 pg_Ru_ cm^–2^_geo_ s^–1^ after 2000 cycles. The LSV results, CV analysis, specific activity, ECSA, and Ru dissolution rates of Ru_0.8_Ti_0.2_O_2_ support that Ru_0.8_Ti_0.2_O_2_ shows improved electrochemical stability compared to RuO_2_ under these conditions. We also find that for both RuO_2_ and Ru_0.8_Ti_0.2_O_2,_ the two-step durability test reveals that the rate of change of activity, ECSA, and Ru dissolution are not the same for 1000 and 2000 cycles, which supports that the surface evolves and affects OER activity, ECSA and Ru dissolution, which underlies the importance of studying the dynamic changes that occur.

As the “stability number” (S-number) provides a useful comparative metric of the relative electrocatalytic activity and electrochemical stability,^137,138^ we calculated the S-number for RuO_2_ and Ru_0.8_Ti_0.2_O_2_ at each testing stage (initial, after 1000 cycles, after 2000 cycles) and calculated a cumulative S-number (Figure 4g,h and Table S4). The S-numbers of RuO_2_ and Ru_0.8_Ti_0.2_O_2_ increase from the initial to 2000 cycles (Figure 4g), which is in line with lower Ru dissolution rates upon cycling (Figure 4e,f). The S-number of RuO_2_ increases after cycling (Figure 4g); however, the mass activity of RuO_2_ decreases with cycling due to the substantial Ru dissolution that occurs (Figure 4e). At each testing stage and cumulatively, the S-numbers for Ru_0.8_Ti_0.2_O_2_ are higher than those of RuO_2_, which supports that Ru_0.8_Ti_0.2_O_2_ exhibits improved electrochemical stability relative to RuO_2_.

We compared our S-numbers with previously reported values^137,138,142^ as well as our own values,^29^ as summarized in Table S4, noting that differences in the materials and electrochemical testing parameters influence the comparison of values. We find that our initial S-number of commercial RuO_2_ (∼10^3^) is within the range of others reported S-values for commercial RuO_2_ (∼10^3^–10^4^), considering differences in materials and electrochemical testing conditions and parameters. The cumulative S-number for Ru_0.8_Ti_0.2_O_2_ (∼10^7^) is higher than values reported for powders of crystalline IrO_2_ (∼10^6^),^137,138^ currently considered as a baseline for OER catalysts that provide a balanced activity and stability, which indicates that after surface reconstruction and initial dissolution, Ru_0.8_Ti_0.2_O_2_ exhibits high electrochemical stability. The importance of considering surface reconstruction, changes over time, and different testing conditions in determining S-numbers are supported by the differences in the S-numbers of our tests of Ru_0.8_Ti_0.2_O_2_ obtained with potential cycling (2000 cycles to 1.8 V_RHE_) and assessed at multiple stages compared to our previously reported S-number for Ru_0.8_Ti_0.2_O_2_ obtained from a single potentiostatic (1.6 V_RHE_ for 13.5 h) accelerated durability test (Table S4).^29^

### 3.6 Electron Microscopy and Spectroscopy Analysis of Structural Changes upon Cycling

**Figure 5:**
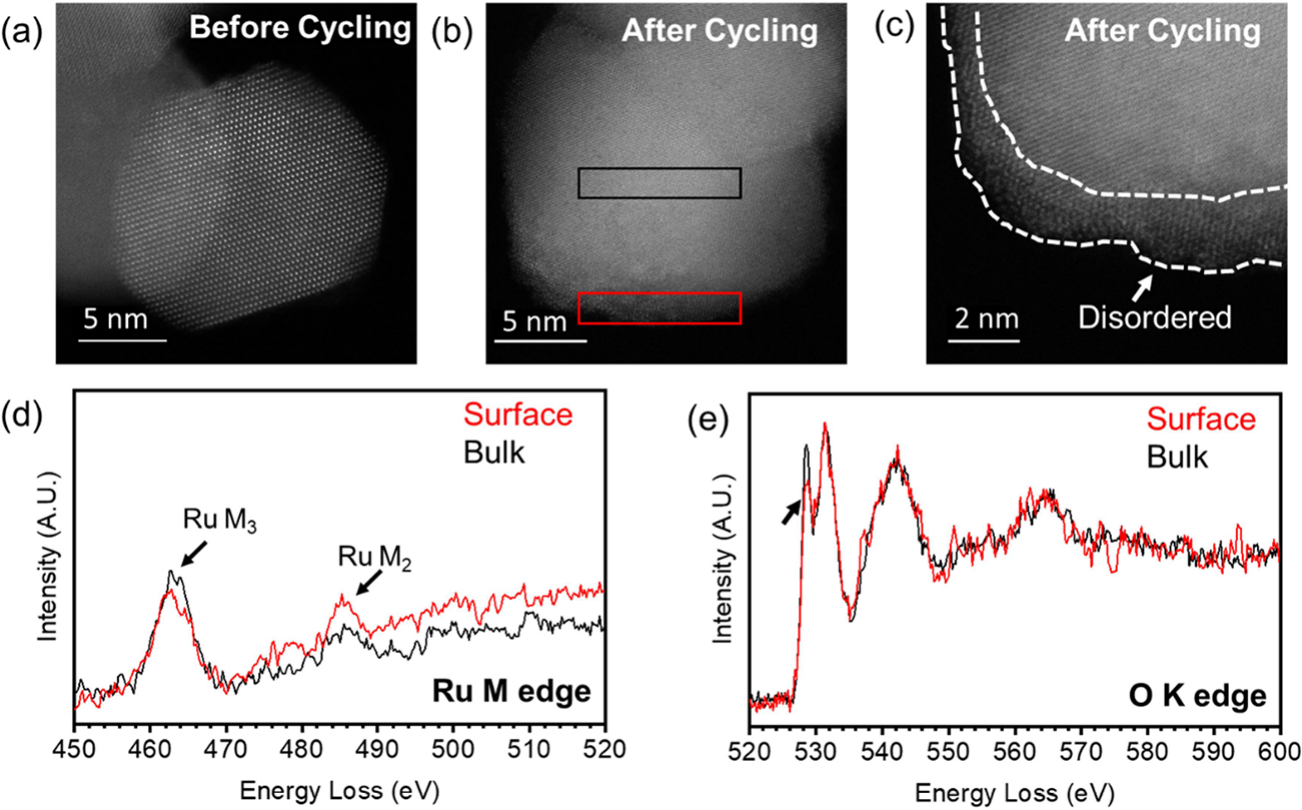
ADF-STEM image of a characteristic RuO_2_ particle before (a) and after (b) cycling, a higher magnification ADF-STEM image (c) of the particle shown in (b), and background subtracted EELS spectra of the Ru M edge (d) and the O K edge (e) comparing the surface (outlined in red in b) vs the bulk (outline in black in b). The dotted white lines in c outline the disordered region at the particle’s surface. The arrow in e highlights the difference in the pre-O K edge peak obtained from the bulk vs the sample’s surface.

To understand how the catalyst surface structure changes upon cycling, we characterized commercial RuO_2_ and our synthesized Ru_0.8_Ti_0.2_O_2_ before cycling and after 2000 cycles using ADF-STEM and EELS, and the results are shown in Figure 5. ADF-STEM images are shown of representative RuO_2_ particles before cycling (Figure 5a) and after cycling (Figure 5b). A higher magnification STEM image (Figure 5c) of the particle after cycling reveals a disordered surface approximately 1–2 nm thick. EELS mapping was performed to see if there was a change in the composition of this disordered surface. The Ru M edge and O K edge EELS spectra from the surface of the particle and the edge of the particle are shown in Figure 5d,e. Notably, the surface spectrum has a different M_3_/M_2_ intensity ratio (Figure 5d) and a less pronounced O prepeak (Figure 5e), suggesting a change in Ru–O bonding within the surface of RuO_2_ after cycling. In contrast, the uncycled RuO_2_ material saw no change in peak intensities between the bulk and the surface of the particle (Figure S12).

**Figure 6:**
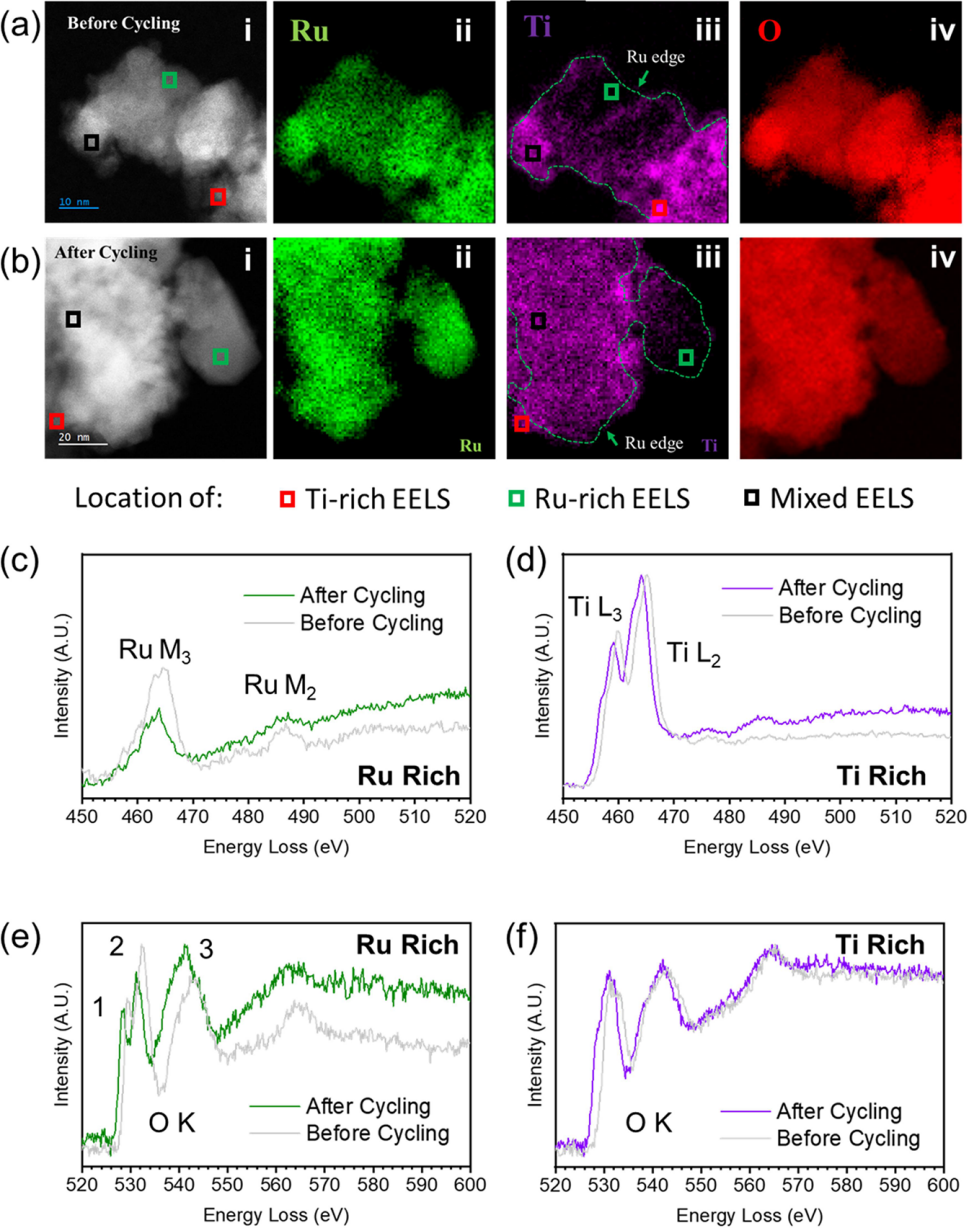
ADF-STEM image of typical Ru_0.8_Ti_0.2_O_2_ particles (i), Ru EELS map (ii), Ti EELS map (iii), and O EELS map (iv) before (a) and after (b) cycling. (c) and (d) Characteristic EELS spectra from Ru-rich (Ru M_2,3_ edge) and Ti-rich (Ti L_2,3_ edge) regions, respectively. (e,f) Characteristic O K edge EELS spectra from the same Ru-rich and Ti-rich regions, respectively. These regions are marked on a-i, a-iii, b-i, and b-iii as red (Ru-rich) or green (Ti-rich) hollow squares. Arrows in (a-iii) and (b-iii) notate Ru edge (green arrows) and Ti-rich regions (purple arrows).

The synthesized Ru_0.8_Ti_0.2_O_2_ particles were also characterized before and after 2000 cycles using ADF-STEM and EELS. Figure 6 shows an ADF-STEM image (i) of representative particles before cycling (Figure 6a) and after cycling (Figure 6b), along with a colored EELS map showing the intensity of Ru (ii), Ti (iii), and O (iv). Given that the Ru M_3_ edge overlaps with the Ti L_2_ edge, as shown in Figures S13a and S14a, the EELS maps were obtained by integrating and plotting the intensity of the Ru N_2,3_, Ti M_2,3,_ and O K edges, respectively. The Ru N_2,3_ and Ti M_2,3_ edges are depicted in Figure S13c.

Our ADF-STEM and EELS mapping analyses of Ru_0.8_Ti_0.2_O_2_ before and after cycling qualitatively support that there are changes to the distribution of Ru and Ti around the edges of particle agglomerates as well as local bonding changes. For the uncycled Ru_0.8_Ti_0.2_O_2_ samples (Figure 6a), Ru was found throughout the particles, although it tended to be most concentrated in the bulk of individual particles. Ti, on the other hand, was primarily found along the surface of particles, being less prominent in the bulk. O was evenly distributed throughout the particles. Although Ti was most rich along the surface of particles, Ru could also be found in these regions, as can be seen with the outline of the Ru map overlaid on the Ti map (Figure 6a-iii). Although it can be observed that more Ti-rich (Ru-poor) regions are present around the edge of the agglomerate after cycling (Figure 6b-iii), the intrinsic heterogeneity and small sample size prevent a preferential loss of Ru at the edge of particles from being definitive. However, analysis of the EELS spectra provides insight into changes in the local bonding environment after cycling.

Analysis of the collected EELS spectra revealed the presence of three types of regions: Ru-rich, Ti-rich, and mixed. Figure 6c–f depicts characteristic core-loss spectra from Ru-rich and Ti-rich regions (Figure S15 depicts a spectrum from a mixed region), which encompasses the Ru M_2,3_, Ti L_2,3,_ and O K edges. The Ru M_3_ edge has a maximum at approximately 464 eV, and the Ru M_2_ edge has a maximum at approximately 486 eV (Figure 6c). The Ti L_3_ and L_2_ edges are doublets centered at approximately 458 and 463 eV (Figure 6d). The O K edge is prevalent in all regions (Figure 6e,f). In Ru-rich regions (Figure 6e), the O K edge closely resembles that of commercial RuO_2_, possessing a prepeak at 529 eV and another peak at 532 eV. However, in Ti-rich regions (Figure 6f), the O K edge lacks this prepeak, suggesting a difference in metal–oxygen bonding in Ti-rich regions. Both Ru-rich and Ti-rich regions possess broad peaks with maxima at approximately 543 and 564 eV.

Following cycling, there is a change in the EELS signature of the Ru-rich regions (Figure 6c). There is a significant decrease in the Ru M_3_ peak intensity contributing to a large change in the M_3_/M_2_ peak ratio. Prior work on other 4*d* transition metals (Mo and Nb) has shown that this peak ratio is related to the transition metal (TM): oxygen ratio as well as the local bonding environment.^143,144^ Furthermore, the Ru core loss spectra after cycling are similar to those observed in the disordered regions of commercial RuO_2_ after cycling. As a reminder, in Figure 5, the edges of RuO_2_ particles became disordered following cycling. The decrease in the Ru M_3_ peak intensity coincided with a change in the Ru M_3_/M_2_ peak ratio (Figure 6c) as well as some minor changes in the O K edge (Figure 6e,f). The similarities between the Ru core loss in the disordered region of commercial RuO_2_ and the Ru-rich region of Ru_0.8_Ti_0.2_O_2_ after cycling suggest that a similar change is occurring in both regions. Meanwhile, the core-loss EELS spectra from Ti-rich regions differ slightly (Figure 6d), with a small shift of both the L_3_ and L_2_ edges to lower energies, which may indicate a slight change in Ti bonding as similar shifts have been reported previously for different Ti oxides.^145^

The O K edge from Ru-rich regions also differs after cycling, with peaks 1, 2, and 3 (as labeled in Figure 6e) shifting to lower energies. Prior work with transition metal oxides has shown that a shift in O K edge peaks can be related to a change in oxidation state.^144^ Meanwhile, the edge of the O K from Ti-rich regions (Figure 6f) is nearly unchanged after cycling. This suggests Ru-rich regions are more susceptible to changes in local bonding structure and TM/O ratios during cycling than those rich in Ti.

### 3.7 Comparison of Theoretical and Experimental Results

Theoretical and experimental analyses of RuO_2_ and Ru_1–*x*_Ti_*x*_O_2_ surfaces show that the oxygen evolution reaction and metal dissolution result in surface reconstruction and changes to the oxygen evolution reaction activity after dissolution. The theory brings new information that currently is not available from experiments. Therefore, a perfect agreement is not expected. Instead, the theory provides a new perspective for atomic-level understanding of this catalytic reaction. Indeed, the comparison of theoretical and experimental results reveals some consistent information and areas for further connection between theory and experiment. From experimental STEM/EELS analysis, we find that electrochemical oxygen evolution and concomitant metal dissolution induce changes to the surface structure (from STEM) and bonding (from EELS, Ru M edge, and Ru K edge changes), as shown in Figures 5 and 6. Computational analysis demonstrates that metal dissolution results in a different arrangement of surface atoms (Figure 3). Metal dissolution changes the metal d-band and the O 2p band centers (Tables 3–5), which agrees with our STEM/EELS analysis showing surface structure and bonding changes resulting from OER and dissolution. For Ru dissolution, the lower Ru dissolution rate from Ru_0.8_Ti_0.2_O_2_ compared to RuO_2_ (Figure 4e,f) is in line with the predicted energy barrier for Ru dissolution being higher in specific sites (e.g., Ru-5C from Ru_0.75_Ti_0.25_O_2_-5D-(110) and Ru_0.75_Ti_0.25_O_2_-6D-(110), shown in Figure 1a,c, compared to Ru-5C from RuO_2_-(110)), which we previously reported.^68^

We also find points of agreement between theory and experiments regarding the effects of metal dissolution and surface reconstruction on the OER activity. From the experimental analysis of the initial materials, RuO_2_ shows a higher specific activity relative to Ru_0.8_Ti_0.2_O_2_ (Figure 4c). Our calculations of the activation energy for the water–oxygen association (Table 6) show that the pristine Ru_0.75_Ti_0.25_O_2_-5D-(110) surface has a higher activation energy (1.89 eV for the Ru site, 2.10 eV for the Ti site) compared to the activation energy for pristine RuO_2_-(110) (1.64 eV); however, we also note that Ru_0.75_Ti_0.25_O_2_-6D-(110) has a lower activation energy (1.48 eV), which indicates the reaction energies are highly site dependent. The experimental materials may have a high degree of Ru_0.75_Ti_0.25_O_2_-5D-(110) surfaces, or differences in experimental and calculated surface sites and other factors may influence the comparison, as discussed further below. Experiments comparing the initial specific activity and that after 1000 cycles (Figures 5c,d) show that metal dissolution and surface reconstruction result in higher OER-specific activity for RuO_2_ and Ru_0.8_Ti_0.2_O_2_. For RuO_2_, our calculations of the water molecule–oxygen association step (Table 6) show that relative to the Ru site for the initial surface, the activation energy barrier decreases for a Ru site from Ru-6C_diss_ from RuO_2_ which is consistent with our experimental data that shows metal dissolution and surface reconstruction result in higher OER specific activity for RuO_2_. Our experimental analysis showed that metal dissolution and surface reconstruction result in higher OER-specific activity for Ru_0.8_Ti_0.2_O_2_, which is in line with our computational analysis of Ru_0.75_Ti_0.25_O_2_-5D-(110) that shows a lower activation energy for a Ru_b_ site after Ru dissolution (Table 6).

The comparison of our theoretical and experimental results also reveals areas for further connection between theory and experiment. Structural differences between the surfaces of the experimental material and our calculated structures influence the comparison of experiment and theory. The experimental surfaces are much more disordered and have heterogeneity compared with our highly ordered calculated surfaces. Our calculations support that the energetic barriers for Ru and Ti dissolution and the OER are highly dependent on the site, further supporting the study of the dynamic surface changes that occur during the OER and metal dissolution. In addition, experimental analysis using operando methods would allow the determination of the actual surface structure under reaction conditions, which remains complex and challenging due to the combination of multiple reaction intermediates, oxygen evolution, metal dissolution, and surface reconstruction that occurs. For computational analysis, further studies with less ordered surfaces would improve connectivity, with experiments showing a disordered surface.

## 4 Conclusions

This work shows that surface reactions and metal dissolution events from ruthenium oxide and ruthenium–titanium oxide electrocatalysts result in surface reconstruction, new surface species, and active sites, and changes in the OER activity and electrochemical stability. From computational analysis, the energy barriers for metal dissolution are highly site-dependent and depend on the Ti-substitution ratio. Ru dissolution results in a solvated RuO_4_ species regardless of the initial configuration, and top-surface titanium tends to vacate the surface, impacting structural stability. Generally, surface reconstruction promoted by the dissolution of metals during cycling generated a more stable structure on both catalysts, with some crucial differences noted in the discussion. After dissolution, significant changes are observed for reactivity indicators such as Ru 4d and O 2p electronic band centers of Ru and O atoms located near surface vacancies obtained upon metal dissolution.

The dissolution of Ru promotes the formation of new active sites with lower activation barriers for the initial reaction (*OOH formation). Conversely, the formation of *OO from *OOH dissociation becomes more challenging, leading to higher activation barriers. Consequently, while O_2_ evolution is generally favored compared to pristine surfaces, it can be hindered due to the reliance on *OO as an intermediate. These results show that in the initial step of the O-water association, the pristine RuO_2_-(110) slab exhibits an activation energy of 1.64 eV, which decreases to 1.48 eV on the Ru_0.75_Ti_0.25_O_2_-6D-(110) slab before metal dissolution. After metal dissolution, particularly Ru-6C dissolution, the activation energy significantly decreases to 0.71 eV on the pristine surface and to 1.38 eV on the Ru_0.75_Ti_0.25_O_2_-5D-(110) slab. The hydroperoxyl oxidation step (*OOH → *OO + H^#^) shows varying performance upon metal dissolution, with activation energy either decreasing to a spontaneous event or increasing compared to the initial state surfaces. Finally, the oxygen released by water adsorption (*OO + H_2_O_(l)_ → O_2(g)_ + H_2_O*) step is improved by the dissolution of both Ru and Ti metals, resulting in lower activation energies in most cases. However, some site-dependent behavior is observed on the Ru_0.75_Ti_0.25_O_2_-5D and -6D slabs after Ru dissolution. Nonetheless, some scenarios exhibit spontaneous *OO formation, suggesting potential variations in the underlying mechanism that require further investigation. In addition to dissolution-induced changes to the surface atoms, our calculations show that electron density changes occur within the solvent within the electrolyte, which underlines the importance of the electrode–electrolyte interface layer’s contribution to the oxygen evolution reaction and metal dissolution reaction.

Experimentally, we determined that the OER-specific activity changes with cycling, and there are significant differences between RuO_2_ and Ru_0.8_Ti_0.2_O_2_. The OER-specific activity of RuO_2_ initially increased and then decreased. In contrast, the OER-specific activity of Ru_0.8_Ti_0.2_O_2_ increases and remains stable upon further cycling. The changes in activity after cycling are consistent with our computational analysis, which shows that the activation barriers for the OER reaction change after metal dissolution. In the case of RuO_2_, changes in the mass activity of RuO_2_ with cycling are directly correlated with Ru dissolution and the lowering of the ECSA. Ru_0.8_Ti_0.2_O_2_ showed a 19 times lower Ru dissolution rate than RuO_2_, and metal dissolution results in increasing the electrochemical surface area and new active sites. Our STEM and EELS analysis support that repeated cycling under OER conditions results in surface reconstruction for RuO_2_ and Ru_0.8_Ti_0.2_O_2_. RuO_2_ particles form a disordered surface (∼1–2 nm thick) after cycling, and EELS analysis supports that Ru–O bonding within the surface is altered compared to that of the pristine material. For Ru_0.8_Ti_0.2_O_2_, metal dissolution induces changes in surface bonding, particularly within Ru-rich regions. Ti-substitution significantly alters metal dissolution, OER activity, and electrochemical stability. Our experimental and computational results both show that exposure to highly oxidative potentials where oxygen evolution and metal dissolution occur results in surface reconstruction and changes to the active catalytic sites and OER activity. Our findings provide insight into the intricate surface atomic and electronic changes induced by metal dissolution and their effect on the oxygen evolution reaction. These multifaceted findings advance our comprehension of metal dissolution dynamics and surface reconstruction and their implications for catalytic processes.

## Supporting Information Available

Additional computational and experimental results and analysis (PDF)
