# Supplementary material for: Impacts of Surface Reconstruction and Metal Dissolution on Ru1–xTixO2 Acidic Oxygen Evolution Electrocatalysts
Source: J Phys Chem C Nanomater Interfaces. 2025 Feb 10;129(7):3595–613. doi: 10.1021/acs.jpcc.4c08119 (PMC11848923; doi:10.1021/acs.jpcc.4c08119)
Supplement: Supplementary file 1 — jp4c08119_si_001.pdf [file jp4c08119_si_001.pdf]

Supporting Information

Impacts of Surface Reconstruction and Metal Dissolution on $Ru_{1-x}Ti_xO_2$ Acidic Oxygen Evolution Electrocatalysts

Francisco A. Ospina-Acevedo,[1,#] José Fernando Godínez-Salomón,[2,#] Zachary G. Naymik,[3] Kevin C. Matthews,[4] Jamie H. Warner,[4,5] Christopher P. Rhodes,[2,3*] and Perla B. Balbuena[1*]

[1] Department of Chemical Engineering, Texas A&M University, College Station, TX 77843, United States

[2] Department of Chemistry and Biochemistry, Texas State University, San Marcos, TX 78666, United States

[3] Materials Science, Engineering and Commercialization Program, Texas State University, San Marcos, TX 78666, United States

[4] Texas Materials Institute, The University of Texas at Austin, Austin, TX, 78712, United States

[5] Walker Department of Mechanical Engineering, The University of Texas at Austin, Austin, TX 78712, United States

*corresponding authors: e-mail: balbuena@tamu.edu; cprhodes@txstate.edu

# These authors contributed equally to this work.

Principal Component Analysis (PCA) of Electron Spectroscopy Data

Principal component analysis (PCA) is a multivariate statistical method that can be used to process data sets. Regarding EELS, PCA can be used to reduce the noise of data sets and identify *principal components* that may correspond to certain features in a sample (for example, Ru-rich regions vs Ti-rich regions). Here, it is primarily used to reduce the noise in the data set. Using HyperSpyUI, the original data sets were decomposed into principal components.[1] These are ranked by the proportion of variance of the data set that is contained in each component. This is often displayed as a scree plot, as shown in Figure S1a. A component index is then selected to determine which components to keep and which components to discard as noise. Often, this component is selected based on the proportion of variance of the components. The proportion of variance of components tends to decrease until it plateaus at some small value. This inflection point is often selected. In the case of Figure S1, that inflection point would be component index 4. However, PCA can also introduce artifacts into the data set or remove *real* information. As such, we opted to select component index 17 for these data sets. Although this may have left some noise, it minimized artifacts and the loss of information. As seen from Figures S1b and S1c, PCA dramatically reduces noise, allowing for easier spectra analysis.

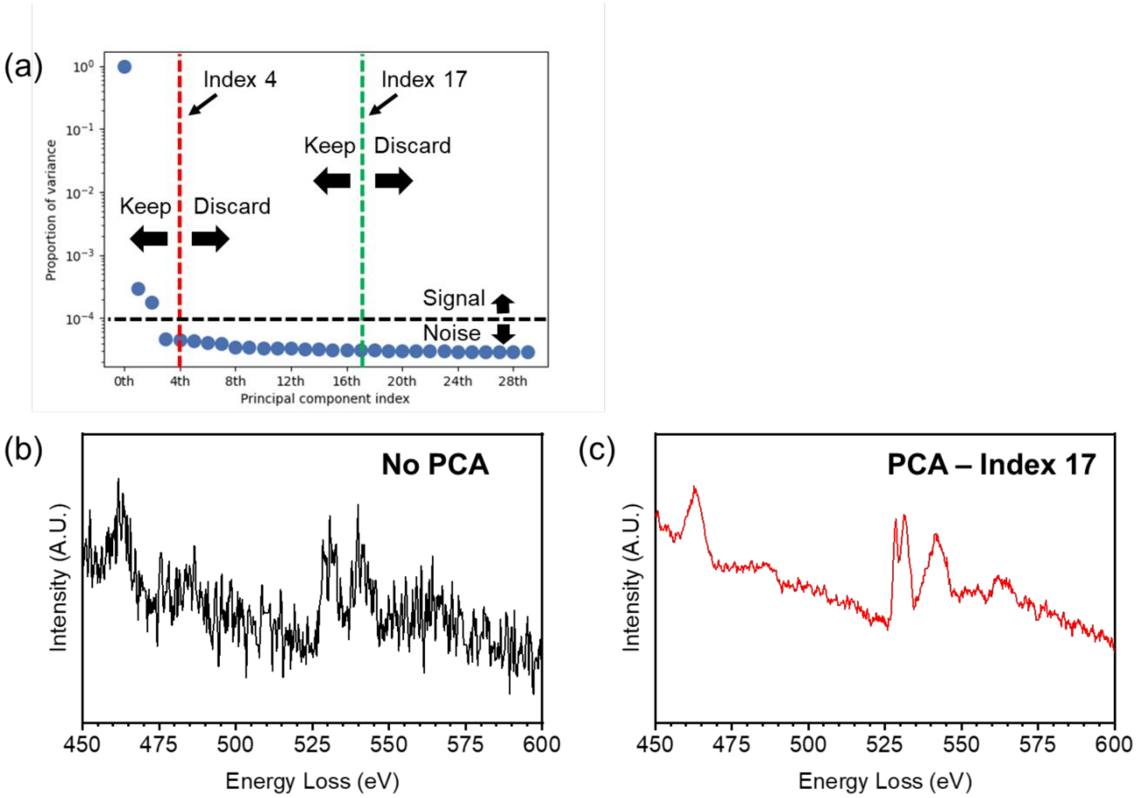

Figure S1. (a) Example screen plot obtained from principal component analysis of a $Ru_{0.8}Ti_{0.2}O_2$ map; the dashed red line indicates the minimum component index that could be selected, the dashed green line indicates the chosen component index that was selected, and the dashed black line represents an approximate cut-off in variance between signal and noise. (b) EELS spectrum obtained from the summation of a 3-pixel-by-3-pixel area without PCA. (c) EELS spectrum obtained from the summation of a 3-pixel-by-3-pixel area with PCA.

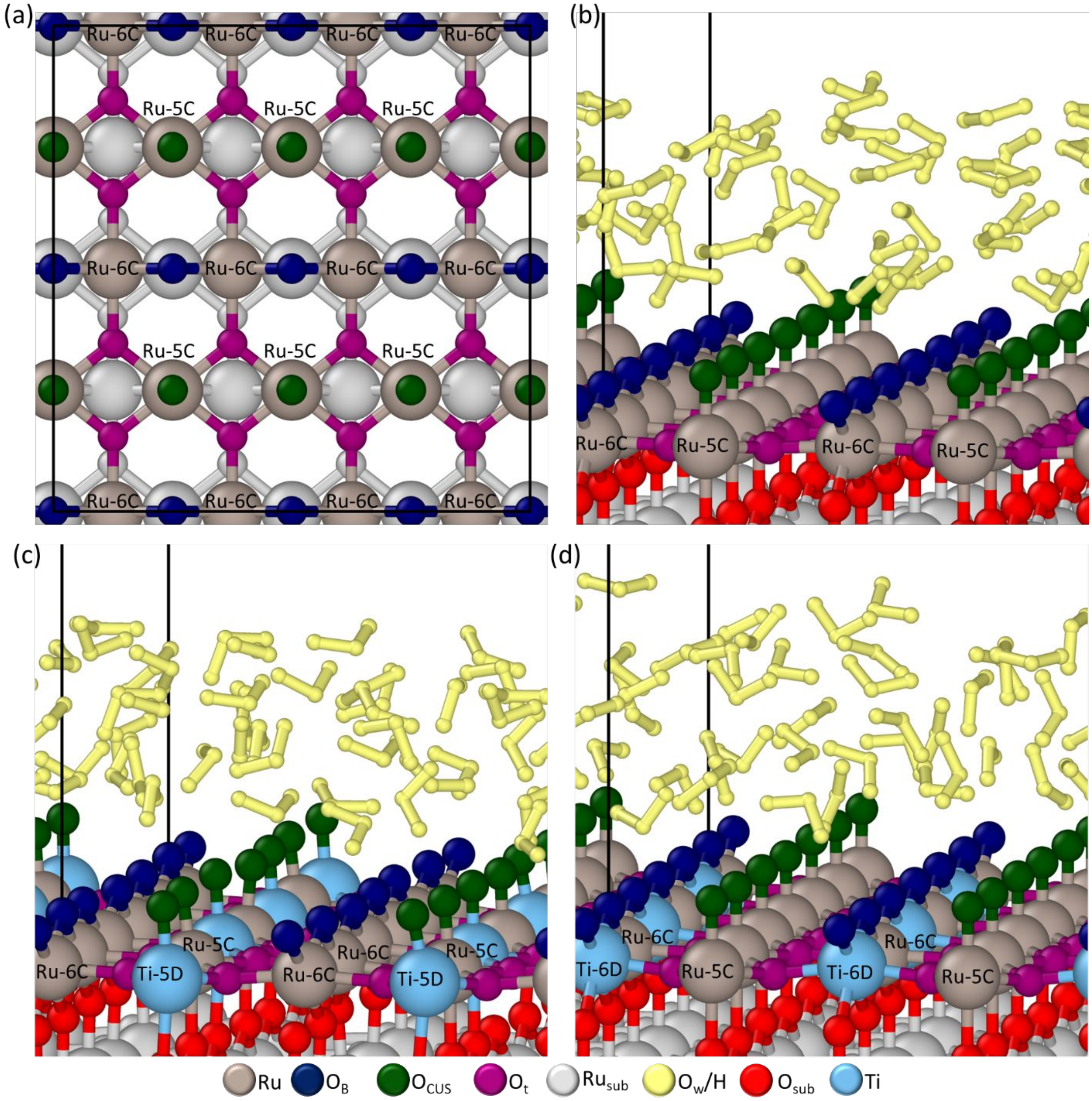

Figure S2. $Ru_{1-x}Ti_xO_2$-(110) surface models used in the theoretical calculations. The surface atoms are shown in colors for better understanding of the different surface atom types on the (a) pristine RuO_2-(110) surface top view, (b) pristine RuO_2-(110) orthogonal view, (c) $Ru_{0.75}Ti_{0.25}O_2$-5D-(110) surface, and (d) $Ru_{0.75}Ti_{0.25}O_2$-6D-(110) surface. Surface ruthenium atoms penta- and hexa-coordinated (Ru-5C and Ru-6C) are shown as large dark grayish spheres; silver spheres are subsurface atoms; surface oxygen atom types are defined as bridging oxygen (O_B, dark blue), coordinatively undersaturated site (O_{CUS}, green), and lattice or tri-coordinate (O_t); water molecules used as explicit solvent are shown in yellow in figures (b) to (d); subsurface oxygen atoms attached to surface metals are shown in red; titanium both in (c) penta- and (d) hexa-coordinated sites are shown in light blue. Figure (a) includes labels for most of the ruthenium sites, while figures (b) to (d) show labels for the first atom of each row for easier identification of the Ru and Ti sites. For reference, the $Ru_{0.50}Ti_{0.50}O_2$-(110) surface is a combination of surfaces shown in (c) and (d), with alternation of both Ti-5D and Ti-6D sites.

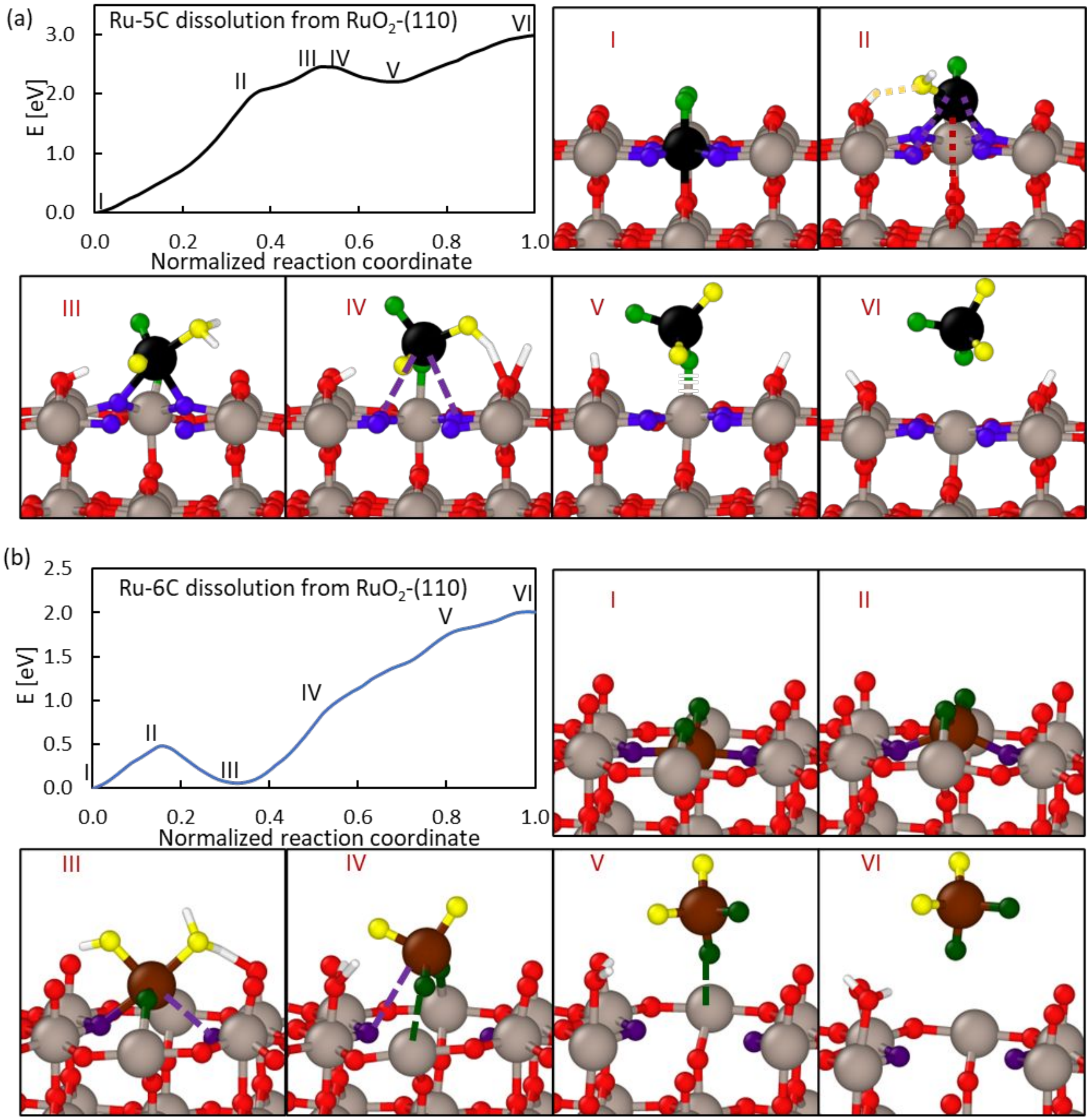

Figure S3. Free energy profile of (a) ruthenium penta- (5C) and (b) hexa-coordinated dissolution from the pristine RuO_2-(110) surface, with the intermediate events along the dissolution path labeled from I (initial state) to VI (Fully dissolved species). Adapted with permission from ref.[2] 2024 ACS Applied Materials & Interfaces, where the intermediate events are explained. Color code: Ru – dark greyish, dissolving Ru – black (Ru-5C) / brown (Ru-6C), O – red, H – white, O_t – purple, O_{CUS} – dark green, O from free water molecule – yellow. Water molecules in the electrolyte are not shown for clarity. Each dissolution pathway exhibits a specific set of intermediate steps, defining unique mechanisms for the Ru-5C and Ru-6C dissolution. Dashed lines represent broken bonds.

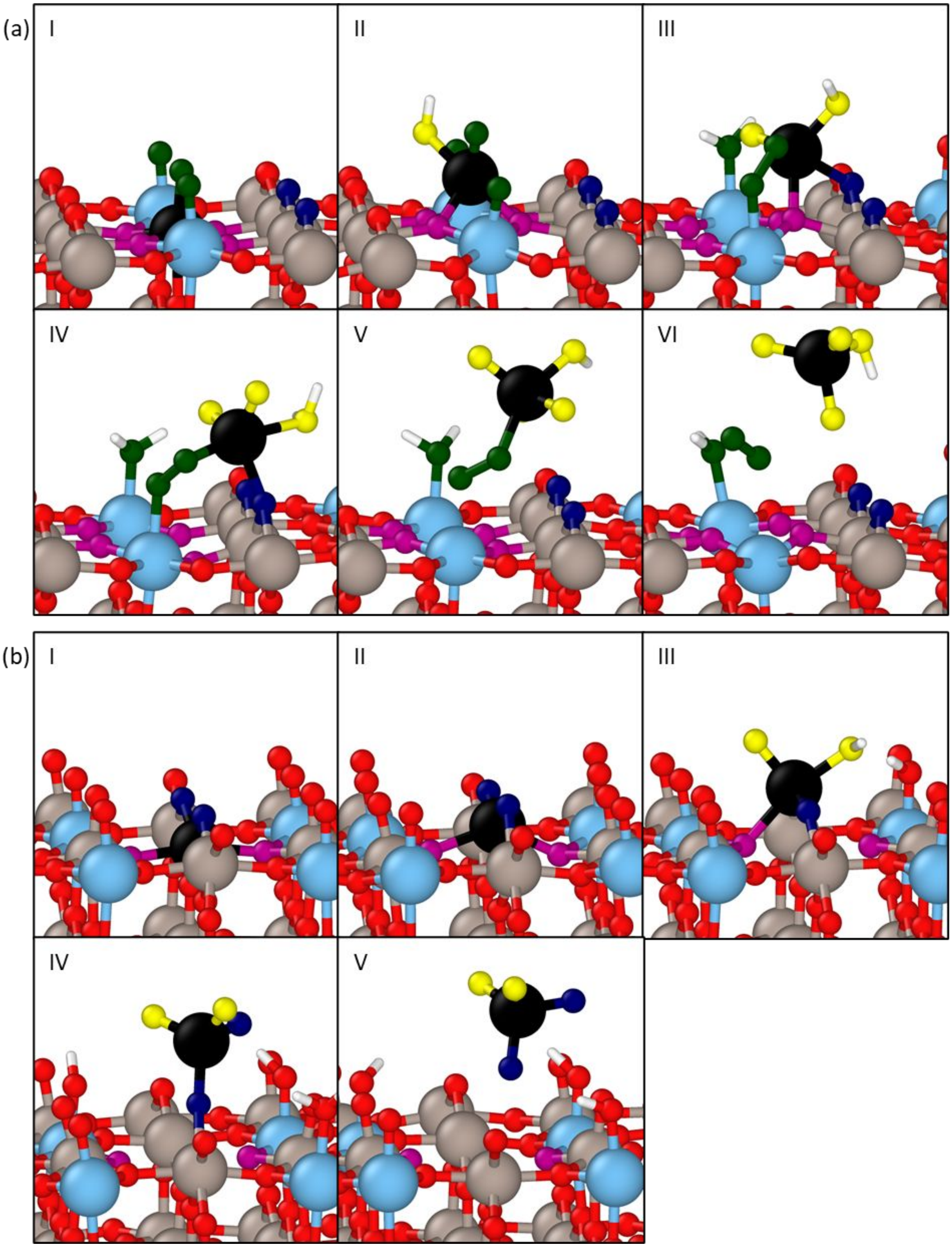

Figure S4. Intermediate events along the dissolution path are labeled from I (initial state) to VI. (Fully dissolved species) from the dissolution of (a) Ru-5C, (b) Ru-6C from the $Ru_{0.75}Ti_{0.25}O_2$-5D-(110) slab. Color code: Ru – silver, Ti – light blue, dissolving Ru – black, O – red, H – white, O_t – purple, O_{CUS} – green, O_B – dark blue, O from free water molecule – yellow.

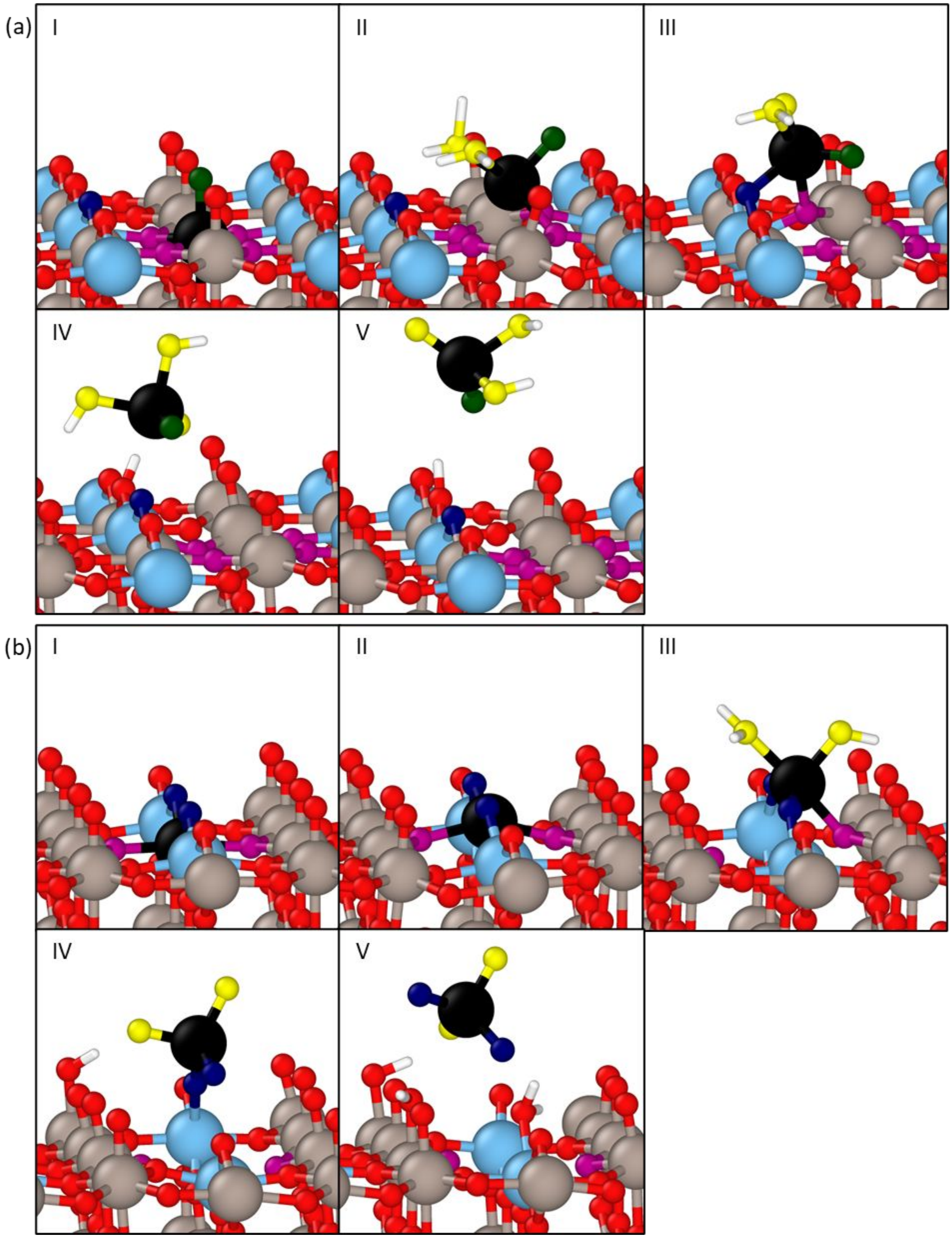

Figure S5. Intermediate events along the dissolution path are labeled from I (initial state) to VI. (Fully dissolved species) from the dissolution of (a) Ru-5C, (b) Ru-6C from the $Ru_{0.75}Ti_{0.25}O_2$-6D-(110) slab. Color code: Ru – silver, Ti – light blue, dissolving Ru – black, O – red, H – white, O_t – purple, O_{CUS} – green, O_B – dark blue, O from free water molecule – yellow.

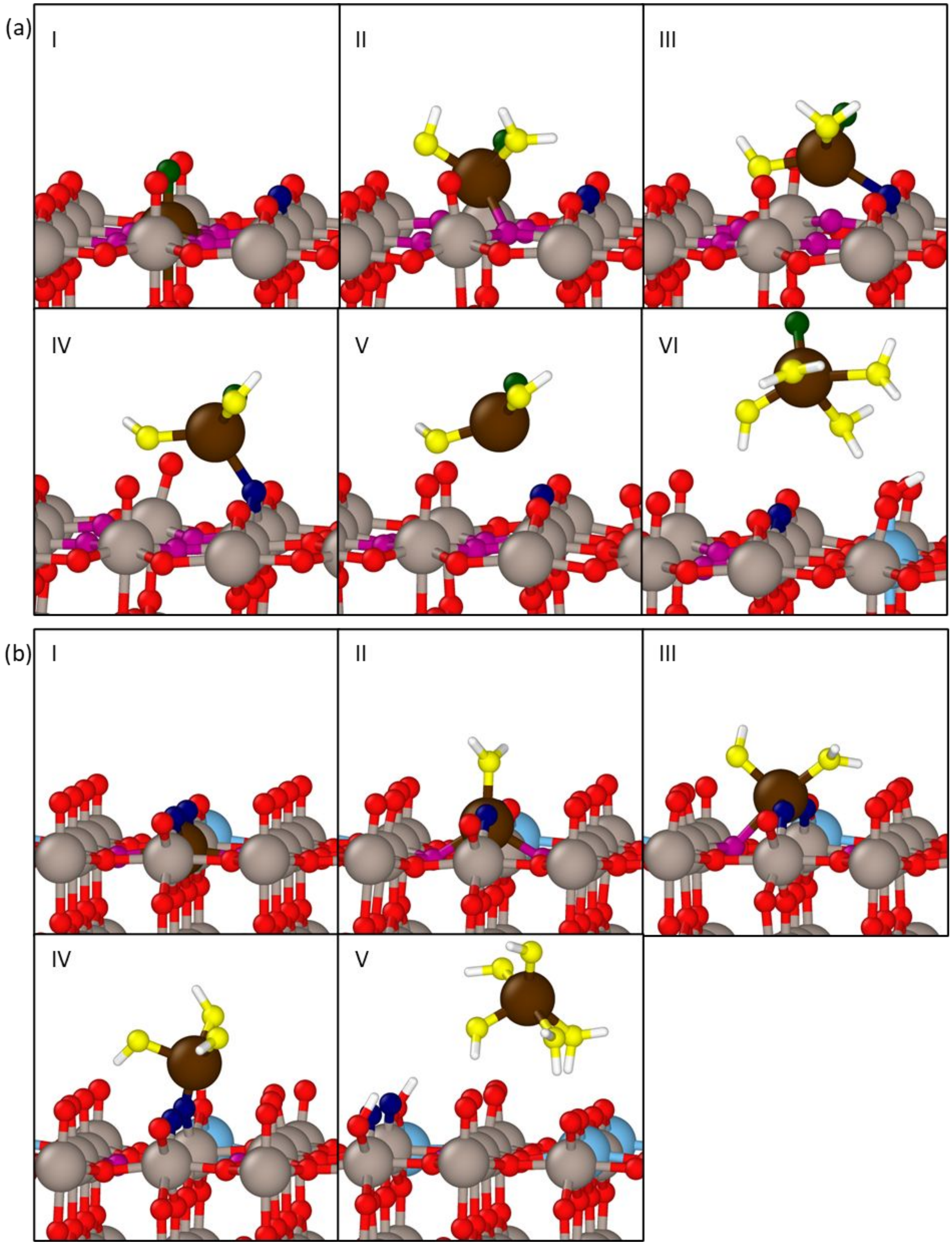

Figure S6. Intermediate events along the dissolution path are labeled from I (initial state) to VI. (Fully dissolved species) from the dissolution of (a) Ti-5D, (b) Ti-6D from the $Ru_{0.75}Ti_{0.25}O_2$-5D and 6D-(110) slabs, respectively. Color code: Ru – dark greyish, Ti – light blue, dissolving Ti – brown, O – red, H – white, O_t – purple, O_{CUS} – green, O_B – dark blue, O from free water molecule – yellow.

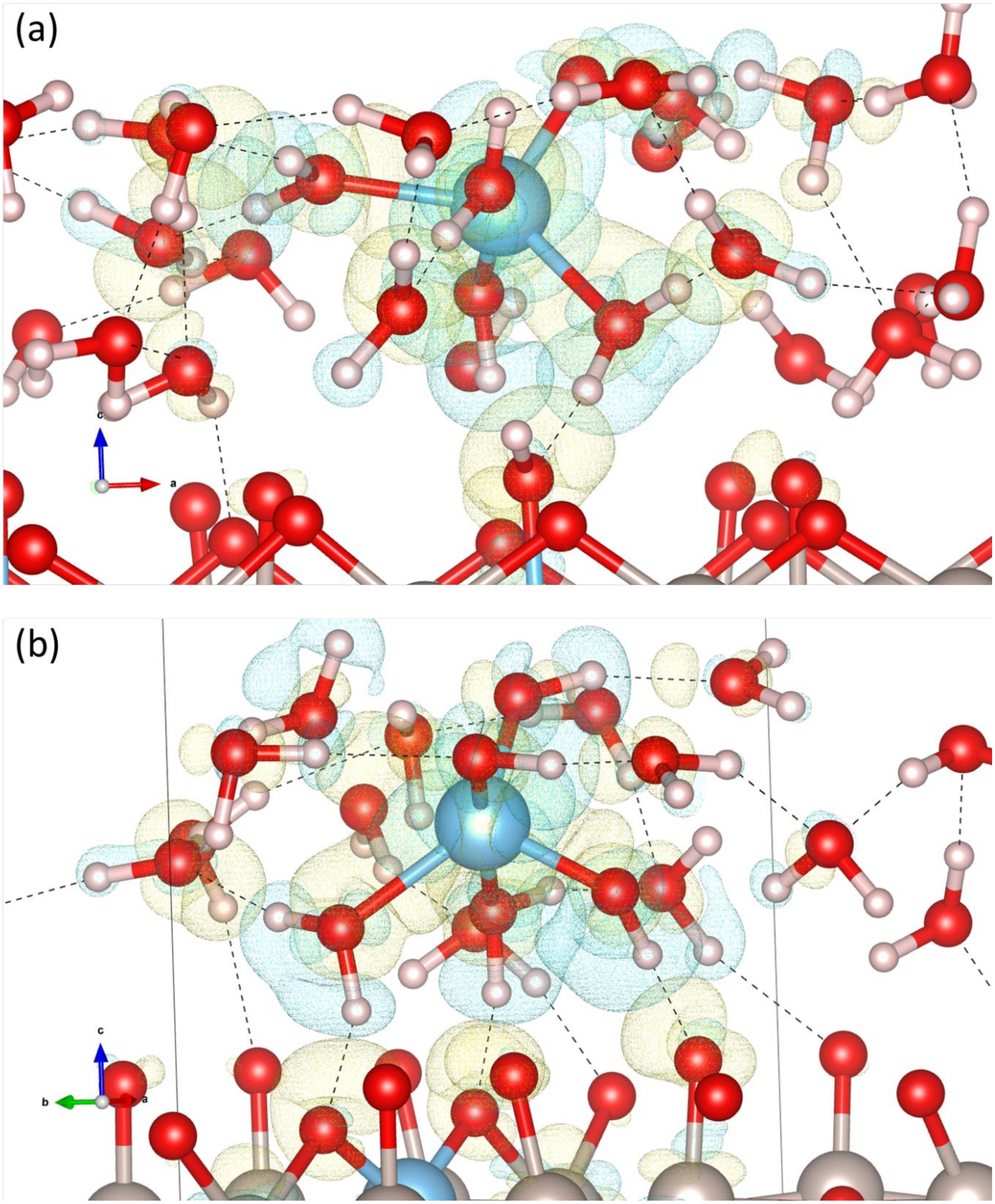

Figure S7. Solvation shell around the Ti-dissolving species from the (a) Ti-5D and (b) Ti-6D dissolution at. 25% Ti substitution. Black dashed lines show the hydrogen bonds in the structures from around 1.68 Å to 2.10 Å, showing the strong H interaction stabilizing the dissolving compounds. The semi-transparent isosurfaces represent the electron density accumulation (yellow) and depletion (blue), showing the complex electronic interaction between dissolving species and surrounding media. More details on the electron density distribution are given in Figure 2 in the main text. Color code: Ru – dark greyish, Ti – light blue, dissolving Ti – brown, O – red, H – light pink.

Computational Analysis of Metal Dissolution at a Higher Concentration of the Substituent: 50% Ti

The effect of a higher Ti concentration on the (110) cleaved surface was evaluated at a 1:1 Ti to Ru ratio. Figure S8 presents the free energy profile for (a, b) Ru and (c, d) Ti dissolution from their initial 5C and 6C configurations, respectively. Figures S9 and S10 show the geometric details for the intermediate states along the dissolution pathways. Table S1 highlights the main events at each step along the free energy pathway. For Ru-5C, the overall process is very costly, suggesting it is unlikely to occur. Ru-6C dissolution energy profile (Figure S8b) exhibits a similar behavior as reported in Figure 1 in the main text for hexa-coordinated metals. Molecular details are shown in Figure S9b. At the last step, based on the different bond lengths of the second O_B atom with Ru (1.76 Å) vs. Ti (2.18 Å), the dissolving compound may be defined as a RuO_4* cluster adsorbed on the new Ti-5D active site. In contrast to Ru-5C, the dissolution of Ru-6C from the 50% substituted Ti surface is much more favorable. Similar behavior was observed in the 25% substituted surfaces, as discussed in the main text and presented in Figure 1. Table S2 provides energetics and details for the events occurring during the dissolution of Ti-5D and Ti-6D. For Ti-5D dissolution, shown in Figure S8c, the free energy profile is similar to the one for Ru-5C dissolution. Still, it requires much less energy and, therefore, is more feasible, and it is possible to expect some Ru enrichment after further electrochemical activity. Molecular details are in Figure S10a. The last case evaluated is the Ti-6D dissolution (S8d), where the free energy profile exhibits two distinctive regions with considerably different slopes. Molecular details are in Figure S10b.

One typical behavior from the 50% Ti-substituted surfaces is that in all cases, a neighbor Ti-6D atom not related to the dissolving metal atom enters the dissolution process by breaking its primary bonds with the subsurface and O_t oxygens attracting and adsorbing free water molecules, as can be seen in the background of some of the states in Figures S9 and S10. This event happens spontaneously, in agreement with the low free energy profile, reinforcing the experimental findings at a 50% concentration of Ti, presenting low structural stability, and forming metal aggregates under phase separation processes. So, even though the results from this concentration show an interesting Ru stabilization after the highest initial activation energies for dissolution, the whole system may not be stable, leading to further Ti dissolution to reach a lower, more stable surface Ti concentration. Another joint event among the different dissolution studies described above is the spontaneous formation of hydroperoxide species due to the oxidation of the electrolyte molecules, which occurs more frequently in the presence of Ti.

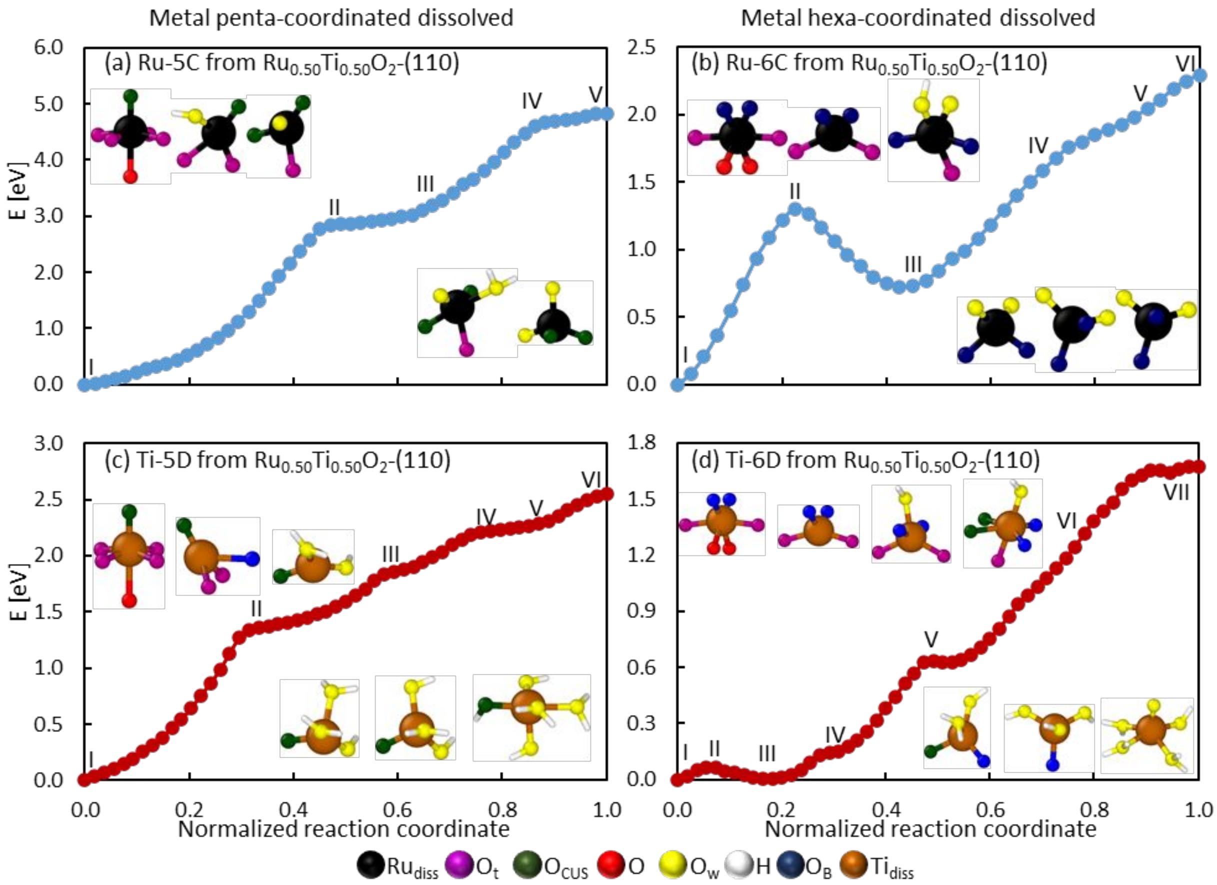

Figure S8. Free energy profile of (a) ruthenium penta- and hexa-coordinated dissolution from the (a) $Ru_{0.50}Ti_{0.50}O_2$-5D-(110) slab, colored blue and (b) titanium penta- (5D) and hexa-coordinated (6D) from the $Ru_{0.50}Ti_{0.50}O_2$-6D-(110) slab, colored red, respectively. The molecular structures are a representation of each estimated state. Color code in Figure.

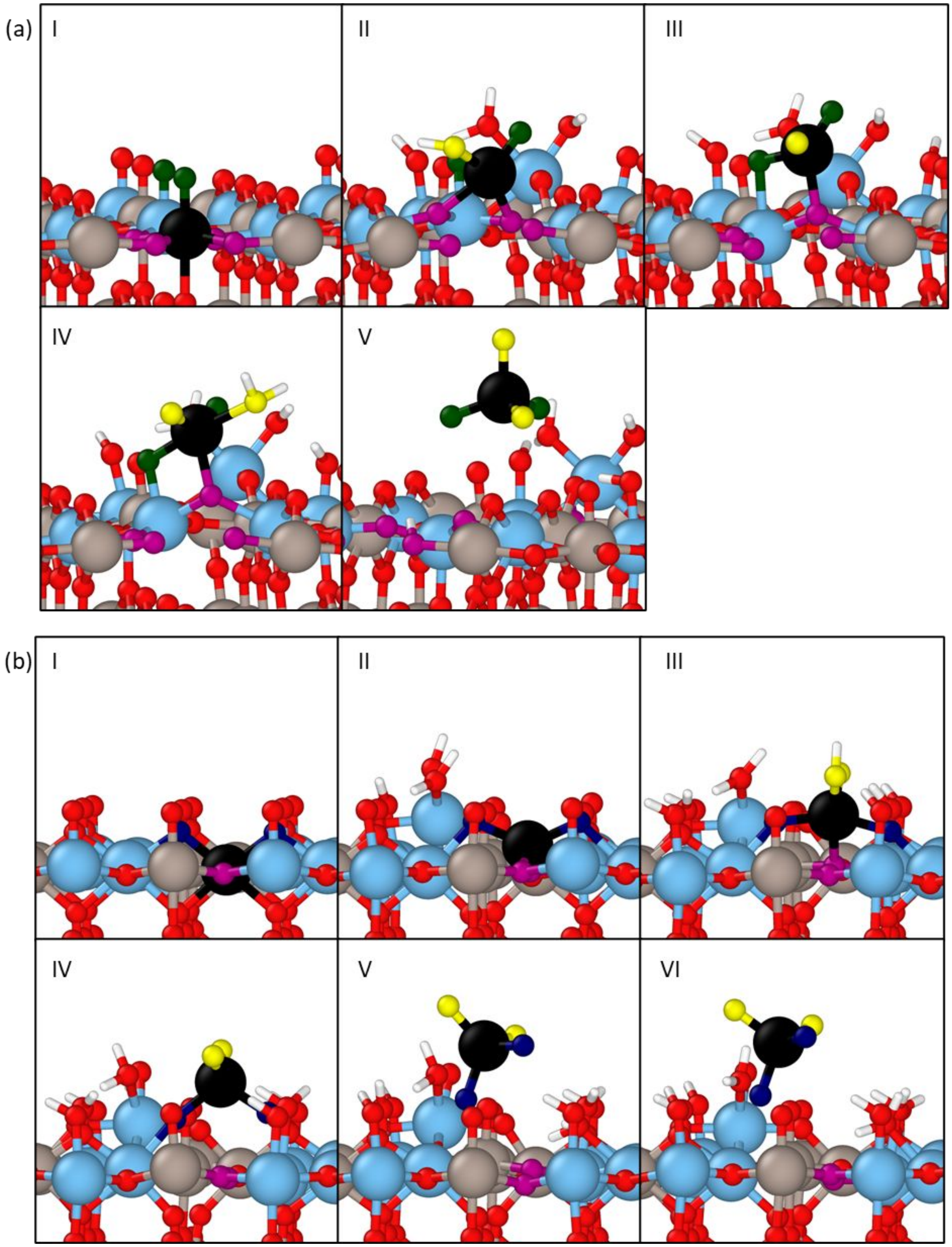

Figure S9. Intermediate events along the dissolution path from the dissolution of (a) Ru-5C and (b) Ru-6C from the $Ru_{0.50}Ti_{0.50}O_2$-(110) slab. Color code: Ru – silver, Ti – light blue, dissolving Ru – black, O – red, H – white, O_t – purple, O_{CUS} – green, O_B – dark blue, O from free water molecule – yellow.

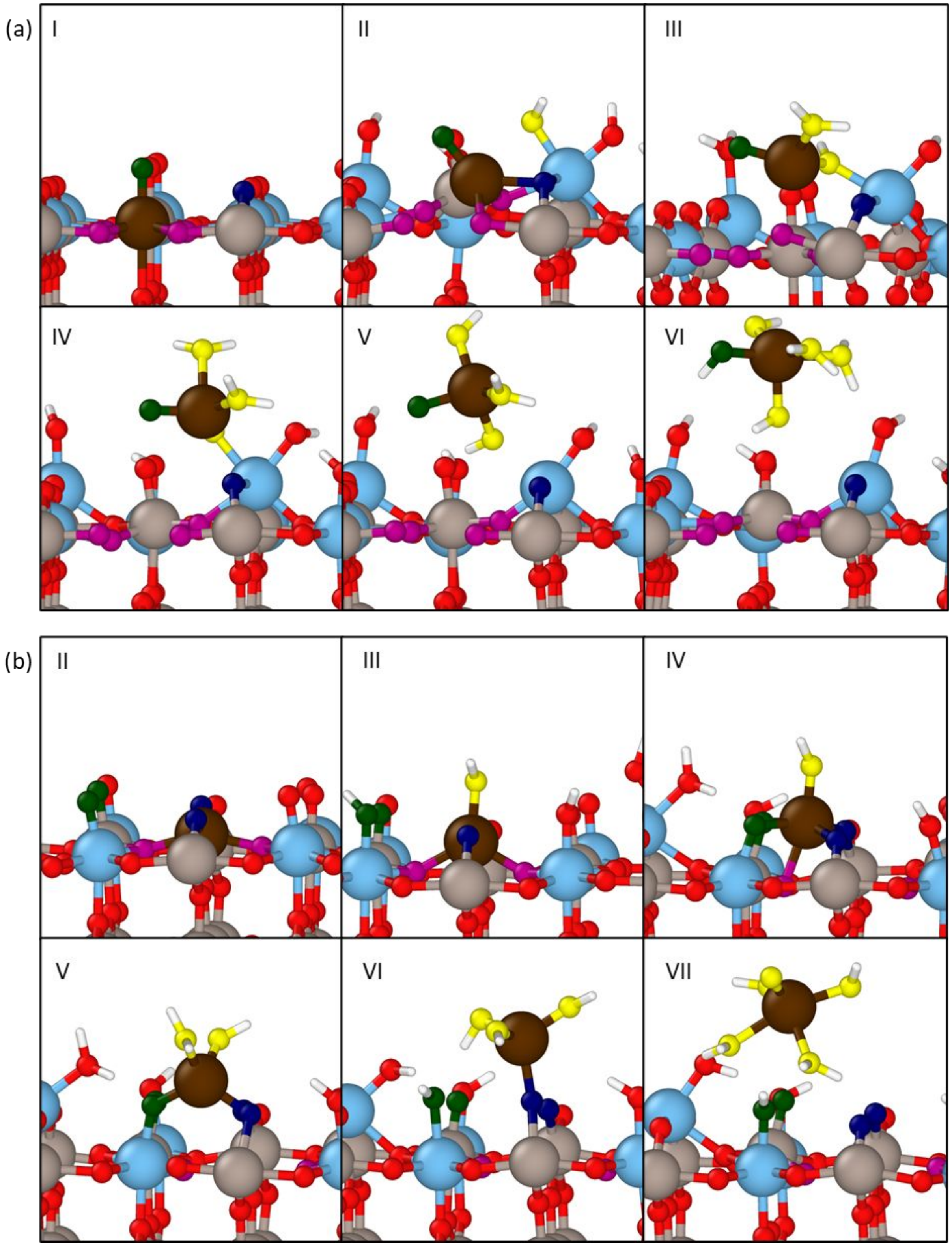

Figure S10. Intermediate events along the dissolution path from the dissolution of (a) Ti-5D and (b) Ti-6D from the $Ru_{0.50}Ti_{0.50}O_2$-(110) slabs. Color code: Ru – silver, Ti – light blue, dissolving Ti – brown, O – red, H – white, O_t – purple, O_{CUS} – green, O_B – dark blue, O from free water molecule – yellow.

Table S1. Dissolution of Ru-5C and Ru-6C from $Ru_{0.50}Ti_{0.50}O_2$ surfaces

Energy barrier (eV)	Event	Physical processes for dissolution of Ru-5C from $Ru_{0.50}Ti_{0.50}O_2$-6D-(110)
2.84	I to II	Ru-subsurface O bond broken, 2 Ru-O_t bonds broken; 1 H_2O molecule adsorbed and partially oxidized
0.18	II to III	Last Ru-O_t bond broken; 1st adsorbed H_2O fully oxidized; bonds to neighbor O_{CUS}.
1.66	III to IV	2nd water adsorbed
0.14	IV to V	Complete oxidation of 2nd water; neighbor O_{CUS} is detached from surface (forming a Ti-5D active site); stable RuO_4 dissolved species
**Energy barrier (eV)**	**Event**	**Physical processes for dissolution of Ru-6C from $Ru_{0.50}Ti_{0.50}O_2$-5D-(110)**
1.30	I to II	Subsurface bonds broken; Ru pushed out of surface
--	II to III	Downhill pathway; Ru-O_t bond broken; 2 H_2O adsorbed (one fully oxidized, and one partially oxidized)
1.04	III to IV	Last Ru-Ot broken; complete oxidation of 2nd water; 1st O_B detached from surface (new type Ti-5D active site created)
0.28	IV to V	2nd O_B detached from surface (creates a 2nd new type Ti-5D active site)
0.25	V to VI	Stable, fully dissolved RuO_4 species

Table S2. Dissolution of Ti-5D and Ti-6D from $Ru_{0.50}Ti_{0.50}O_2$ surfaces

Energy barrier (eV)	Event	Physical processes for dissolution of Ti-5D from $Ru_{0.50}Ti_{0.50}O_2$-6D-(110)
1.34	I to II	Ti-subsurface O bond broken; Ti-O_t bond broken; dissolving Ti-O_B bond formed and pulls neighbor Ti-6D.
0.49	II to III	Last Ti-O_t bonds broken; Ti detaches from $O_{B;}$ one water adsorbed; one water partially oxidized shared with neighbor Ti-6D that becomes part of the dissolving cluster
0.37	III to IV	Adsorption of 2nd water; neighbor Ti-6D keeps bonded to O_B, O_t, and to the dissolving cluster via the shared partially oxidized water molecule
0.16	IV to V	Partially oxidation of 2nd water molecule adsorbed; dissolving cluster separates from neighbor Ti, keeping the previously shared HO* group.
0.20	V to VI	Dissolving cluster stabilizes as TiO_5H_7 dissolved species
**Energy barrier (eV)**	**Event**	**Physical processes for dissolution of Ti-6D from $Ru_{0.50}Ti_{0.50}O_2$-5D-(110)**
0.07	I to II	Subsurface bonds broken
--	II to III	Downhill pathway; one water adsorbed and partially oxidized (Ti acts as a 5D active site)
0.15	III to IV	Ti-O_t bond broken; Ti-6D coordinates with O_{cus} adsorbed on Ru-5C, and with another O_{cus} adsorbed on Ti-5D
0.49	IV to V	Ti-6D breaks bonds with one O_B, with O_t, and with one O_{cus}; adsorbs 2nd H_2O
0.67	V to VI	Breaks 2nd O_{cus} bond; adsorbs a 3rd H2O and partially oxidize 2nd and 3rd H_2O
0.35	VI to VII	Breaks last O_B bond (creates two new type of oxidized Ru-5C sites); oxidizes one HO* and adsorbs two H_2O; dissolves in aqueous media as $TiO_5H_{7.}$

Cyclic Voltammetry and Electrochemical Surface Area (ECSA) of RuO_2 and $Ru_{0.8}Ti_{0.2}O_2$

Cyclic voltammograms (CVs) of RuO_2 and $Ru_{0.8}Ti_{0.2}O_2$ are shown in Figure S11. In general, the CVs of both catalyst materials showed broad oxidation/reduction peaks from 0.4 to 1.0 V, consistent with pseudocapacitive charge storage,[3] and an additional reduction peak below 0.3 V. Pseudocapacitance is characteristic of hydrous RuO_2 (general formula RuO_xH_y or $RuO_2{\cdot}xH_2O$) which has a disordered surface region.[4] Hydrous RuO_2 can allow charge storage below the top surface layer, which results in higher specific capacitance and area-normalized capacitance values as compared with anhydrous RuO_2.[3, 5] Using the specific capacitances and BET surface areas, we determined area-normalized capacitance values of RuO_2 and $Ru_{0.8}Ti_{0.2}O_2$ (Table S3). The area-normalized capacitance values of RuO_2 (173 μF cm^{-2}) and $Ru_{0.8}Ti_{0.2}O_2$ (25 μF cm^{-2}) are much higher than the value reported for anhydrous RuO_2 (2 μF cm^{-2}),[3] which

supports that the surfaces of these materials more closely resemble that of hydrous RuO_2 than anhydrous RuO_2. In addition, RuO_2 and $Ru_{0.8}Ti_{0.2}O_2$ have different area-normalized capacitance values, which indicates that surfaces may have different degrees of structural hydration within the surface layer. Given the hydrous character of the surfaces and the limitations of using double-layer capacitance values with disordered RuO_2 surfaces,[6] we used the area-normalized capacitance values to determine the electrochemical surface area (ECSA) of the RuO_2 and $Ru_{0.8}Ti_{0.2}O_2$.

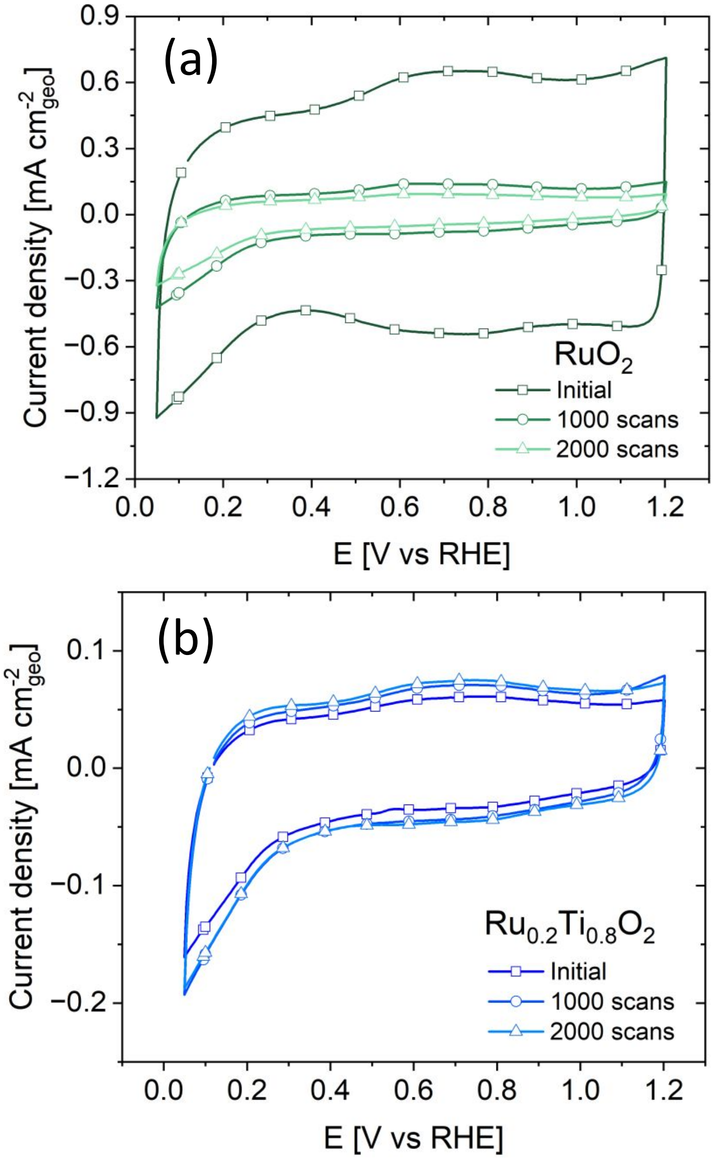

Figure S11. Cyclic Voltammograms of RuO_2 (a) and $Ru_{0.8}Ti_{0.2}O_2$ (b), initial, after 1000 cycles and after 2000 cycles in 0.1 M $HClO_4$ using a scan rate of 100 mV s^{-1}.

Table S3. Brunauer, Emmett, and Teller (BET) surface area, specific capacitance (C_s), areal capacitance (Capacitance/$Area_{BET)}$ and electrochemical surface area (ECSA) values of commercial RuO_2 and $Ru_{0.8}Ti_{0.2}O_2$. As previously reported, area-normalized capacitance values were determined from the specific capacitance and BET surface area.[3]

	BET surface area [m^2 g^{-1}]		Specific capacitance C_s [F g^{-1}]		Capacitance/$Area_{BET}$ C_{area} [μF/cm^2]		ECSA [m^2 g^{-1}cat]	
	**RuO_2**	**$Ru_{0.8}Ti_{0.2}O_2$**	**RuO_2**	**$Ru_{0.8}Ti_{0.2}O_2$**	**RuO_2**	**$Ru_{0.8}Ti_{0.2}O_2$**	**RuO_2**	**$Ru_{0.8}Ti_{0.2}O_2$**
**Initial**	66 ± 2	36 ± 4	111 ± 6	9 ± 1	173 ± 10	25 ± 1	64 ± 4	36 ± 1
**1000 cycles**	–	–	20 ± 3	11 ± 1	173 ± 10	25 ± 1	12 ± 2	44 ± 4
**2000 cycles**	–	–	13 ± 3	11 ± 1	173 ± 10	25 ± 1	8 ± 1	46 ± 2

Scanning transmission electron microscopy (STEM) and electron energy loss spectroscopy (EELS)

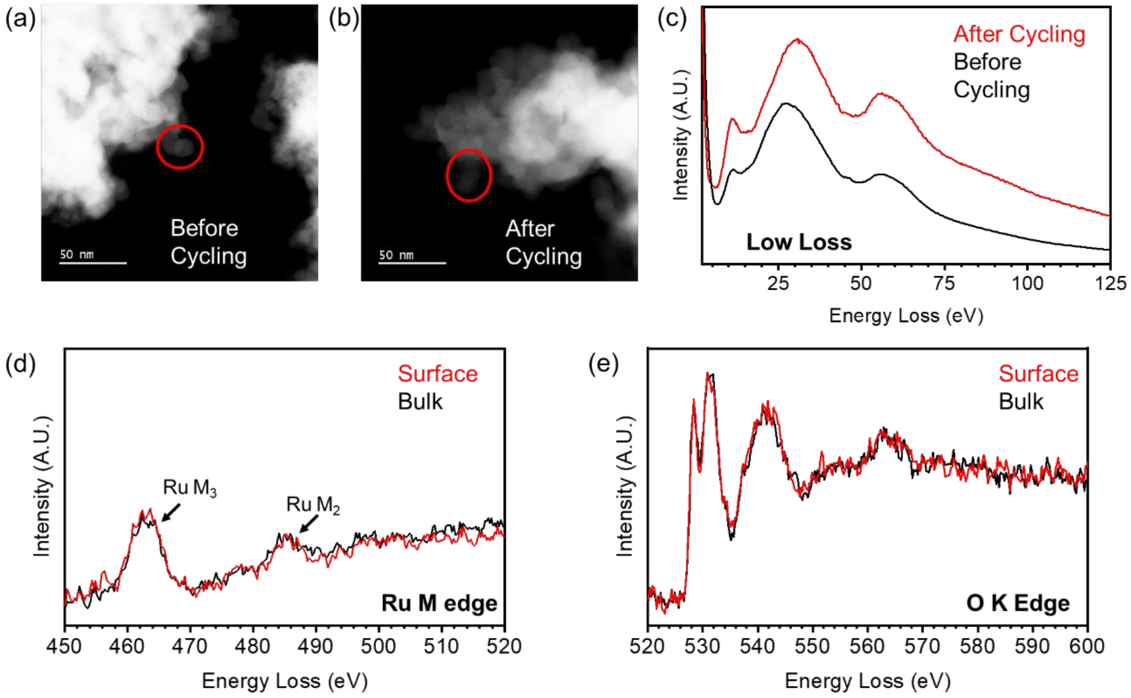

Figure S12. ADF-STEM images showing clusters of RuO_2 particles before (**a**) and after (**b**) cycling; the particles circled in red are those shown in main text Figure 7. A low-loss EELS spectrum from the bulk of a RuO_2 particle before and after cycling (c). Characteristic Ru M edge (d) and O K edge (e) EELS spectra from a RuO_2 particle before cycling from both the bulk and surface of the particle.

EELS mapping of the $Ru_{0.8}Ti_{0.2}O_2$ samples revealed Ru-rich, Ti-rich, and mixed regions. Characteristic EELS spectra for Ru-rich, Ti-rich, and mixed regions are shown in Figures S13 (before cycling) and S14 (after cycling). Prominent Ru M_3 and M_2 edges (Figure S13a) identify Ru-rich regions along with the lack of a Ti L_3 edge and Ti $M_{2,3}$ edge (Figure S13c). Ti-rich regions are identified by prominent Ti L_3 and L_2 edges along with an absent or less intense Ru M_2 edge (Figure S13a). The Ti L_2 and Ru M_3 edges overlap, leaving the Ti L_3, Ru M_2, Ti $M_{2,3,}$ and Ru $N_{2.3}$ edges as the best ways to distinguish Ru-rich and Ti-rich regions. Additionally, the O K edge also differs between Ru-rich and Ti-rich regions (Figure S13b). Ru-rich regions possess a pre-peak before the O K edge at ~529 eV, while Ti-rich regions lack this peak, as seen in Figures S13b and S14b. This suggests that there is a difference in Ru-O and Ti-O bonding.

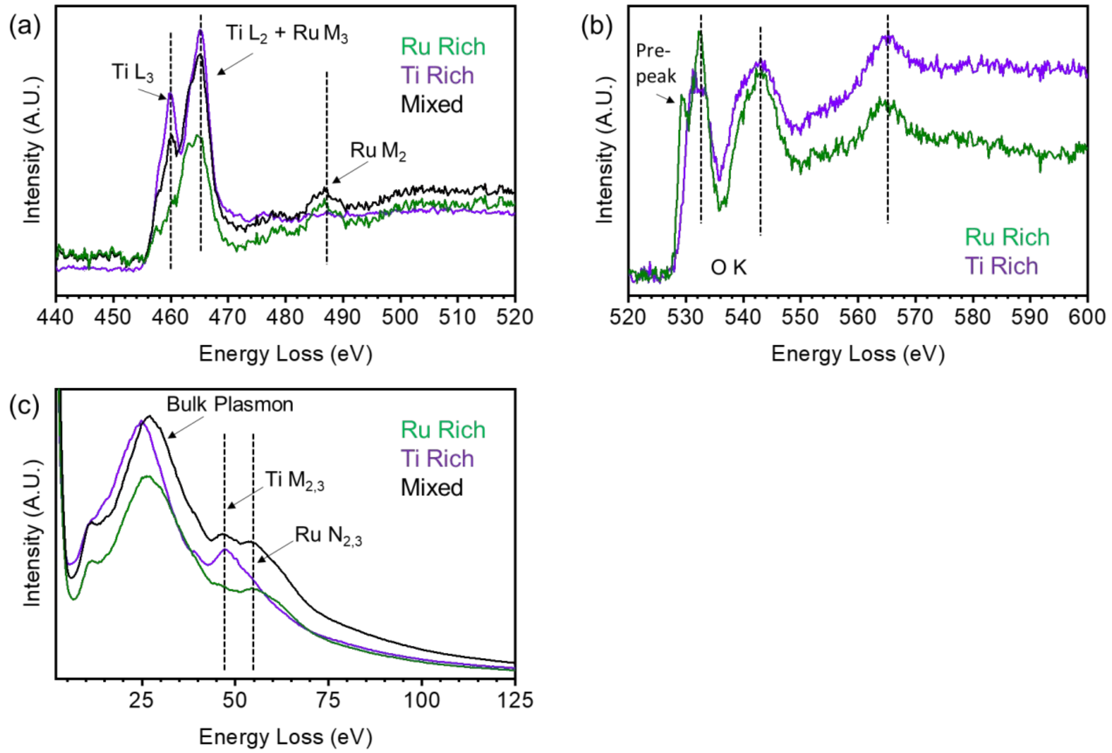

Figure S13. Before Cycling: a) Background subtracted Ru M edge / Ti L edge EELS spectra from a Ru-rich, Ti-rich, and mixed region, b) Background subtracted O K edge EELS spectra from a Ru-rich and a Ti-rich region, and c) Low loss EELS spectra from a Ru rich, Ti-rich, mixed region.

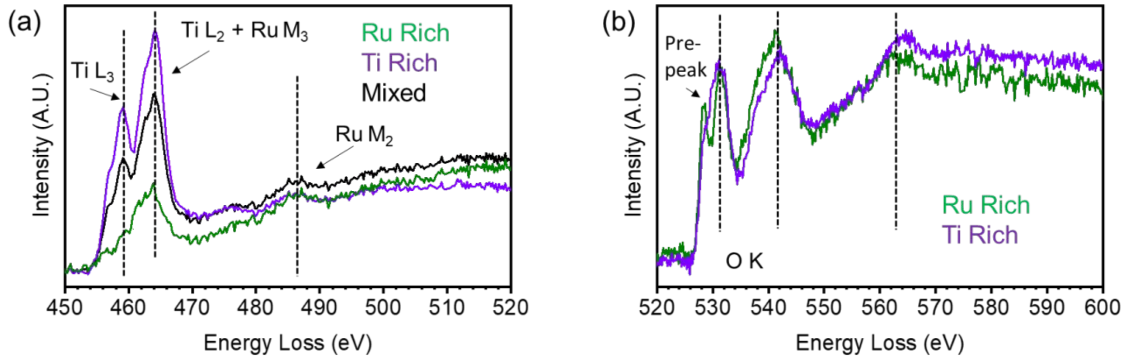

Figure S14. After Cycling: a) Background subtracted Ru M edge / Ti L edge EELS spectra from a Ru-rich, Ti-rich, and mixed region, b) Background subtracted O K edge EELS spectra from a Ru-rich and a Ti-rich region.

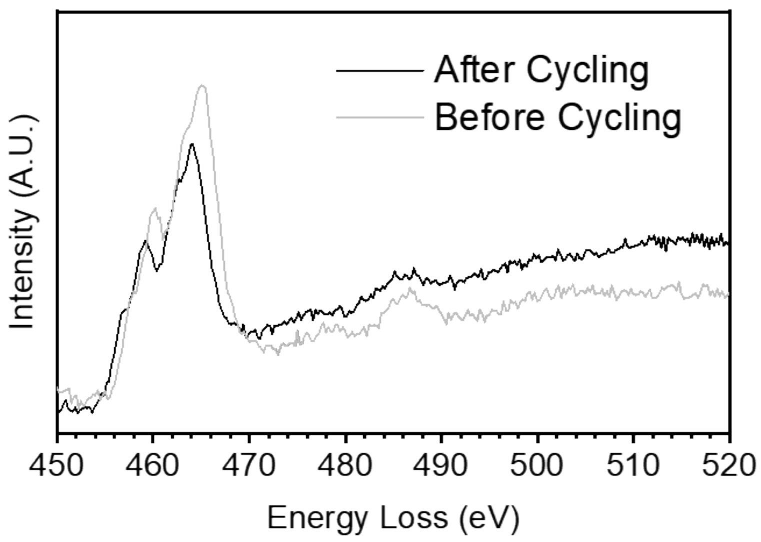

Figure S15. Background subtracted Ru M edge / Ti L edge EELS spectra from a mixed region before and after cycling.

Table S4. Comparison of stability number (S-number) for Ru- and Ir-based OER catalysts from this work and prior studies. “LSV” notates linear sweep voltammetry.

Material	S-number, initial	S-number, 1000 cycles	S-number, 2000 cycles	Cumulative S-number	Accelerated durability testing parameters	Ref.
RuO_2, commercial (Alfa)	4.4 ± 2.2 x 10^3	4.3 ± 0.3 x 10^6	1.9 ± 0.1 x 10^7	5.6 ± 0.5 x 10^6	2000 cycles (1.3 V_{RHE} to 1.8 V_{RHE}) in Ar-purged 0.1 M $HClO_4$	This Work
$Ru_{0.80}Ti_{0.20}O_2$, hydrothermal	3.9 ± 2.1 x 10^4	4.0 ± 2.8 x 10^7	4.9 ± 2.3 x 10^7	3.7 ± 2.3 x 10^7	2000 cycles (1.3 V_{RHE} to 1.8 V_{RHE}) in Ar purged 0.1 M $HClO_4$	
RuO_2, hydrothermal	–	–	–	9.1 ± 2.0 x 10^5	1.6 V_{RHE} for 13.5 h in Ar-purged 0.1 M $HClO_4$	7
$Ru_{0.80}Ti_{0.20}O_2$, hydrothermal	–	–	–	1.8 ± 0.5 x 10^5	1.6 V_{RHE} for 13.5 h in Ar-purged 0.1 M $HClO_4$	
IrO_2 film	–	–	–	2.5 ± 0.5 x 10^5	LSV (1.8 V_{RHE}) in Ar-purged 0.1 M $HClO_4$	8
IrO_2 powder	–	–	–	1.9 ± 1.3 x 10^6	LSV (1.8 V_{RHE}) in Ar-purged 0.1 M $HClO_4$	
IrO_x powder	–	–	–	5.6 ± 0.7 x 10^4	LSV (1.65 V_{RHE}) in Ar-purged 0.1 M $HClO_4$	
Ba_2PrIrO_6 powder	–	–	–	1.7 ± 0.5 x 10^4	LSV (1.65 V_{RHE}) in Ar-purged 0.1 M $HClO_4$	
Ba_2YIrO_6 powder	–	–	–	9.0 ± 2.0 x 10^3	LSV (1.65 V_{RHE}) in Ar-purged 0.1 M $HClO_4$	
Sr_2YIrO_6 powder	–	–	–	5.9 ± 8.0 x 10^3	LSV (1.65 V_{RHE}) in Ar-purged 0.1 M $HClO_4$	
RuO_2, commercial, (Sigma Aldrich)	–	–	–	1.39 x 10^4	13 h at 10 mA cm^{-2} in O_2 saturated 0.1 M $HClO_4$	9
Si-RuO_2	–	–	–	3.42 x 10^4	13 h at 10 mA cm^{-2} in O_2 saturated 0.1 M $HClO_4$	
RuO_2 powder	-–	-–	-–	3.95 x 10^3	13 h at 10 mA cm^{-2} in O_2 saturated 0.1 M $HClO_4$	
RuO_2, Commercial (Sigma Aldrich)	–	–	–	1.0 x 10^3	1.56 V_{RHE} for 10 h in O_2-purged 0.1 M $HClO_4$	10
IrO_2 commercial	–	–	–	5.0 x 10^6	1.56 V_{RHE} for 10 h in O_2-purged 0.1 M $HClO_4$	
IrO_x powder	–	–	–	1.0 x 10^5	1.56 V_{RHE} for 10 h in O_2-purged 0.1 M $HClO_4$	

References

1. Cueva, P.; Hovden, R.; Mundy, J. A.; Xin, H. L. L.; Muller, D. A., Data Processing for Atomic Resolution Electron Energy Loss Spectroscopy. *Microsc. Microanal.* **2012,** *18* (4), 667-675.

2. Ospina-Acevedo, F.; Albiter, L. A.; Bailey, K. O.; Godínez-Salomón, J. F.; Rhodes, C. P.; Balbuena, P. B., Catalytic Activity and Electrochemical Stability of Ru1–xMxO2 (M = Zr, Nb, Ta): Computational and Experimental Study of the Oxygen Evolution Reaction. *ACS Applied Materials & Interfaces* **2024,** *16* (13), 16373-16398.

3. Long, J. W.; Swider, K. E.; Merzbacher, C. I.; Rolison, D. R., Voltammetric characterization of ruthenium oxide-based aerogels and other RuO_2 solids: The nature of capacitance in nanostructured materials. *Langmuir* **1999,** *15* (3), 780-785.

4. McKeown, D. A.; Hagans, P. L.; Carette, L. P. L.; Russell, A. E.; Swider, K. E.; Rolison, D. R., Structure of hydrous ruthenium oxides: Implications for charge storage. *Journal of Physical Chemistry B* **1999,** *103* (23), 4825-4832.

5. Zheng, J. P.; Cygan, P. J.; Jow, T. R., Hydrous Ruthenium Oxide and an Electrode Material for Electrochemical Capacitors. *J. Electrochem. Soc.* **1995,** *142* (8), 2699-2703.

6. Reiser, C.; Kessler, P.; Kamp, M.; Jovic, V.; Moser, S., Specific Capacitance of RuO2(110) Depends Sensitively on Surface Order. *J. Phys. Chem. C* **2023,** *127* (7), 3682-3688.

7. Godínez-Salomón, J. F.; Ospina-Acevedo, F.; Albiter, L. A.; Bailey, K. O.; Naymik, Z. G.; Mendoza-Cruz, R.; Balbuena, P. B.; Rhodes, C. P., Titanium Substitution Effects on the Structure, Activity, and Stability of Nanoscale Ruthenium Oxide Oxygen Evolution Electrocatalysts: Experimental and Computational Study. *ACS Applied Nano Materials* **2022,** *5* (8), 11752-11775.

8. Geiger, S.; Kasian, O.; Ledendecker, M.; Pizzutilo, E.; Mingers, A. M.; Fu, W. T.; Diaz-Morales, O.; Li, Z.; Oellers, T.; Fruchter, L.; Ludwig, A.; Mayrhofer, K. J. J.; Koper, M. T. M.; Cherevko, S., The stability number as a metric for electrocatalyst stability benchmarking. *Nature Catalysis* **2018,** *1* (7), 508-515.

9. Ping, X.; Liu, Y.; Zheng, L.; Song, Y.; Guo, L.; Chen, S.; Wei, Z., Locking the lattice oxygen in RuO2 to stabilize highly active Ru sites in acidic water oxidation. *Nature Communications* **2024,** *15* (1), 2501.

10. Hubert, M. A.; Patel, A. M.; Gallo, A.; Liu, Y.; Valle, E.; Ben-Naim, M.; Sanchez, J.; Sokaras, D.; Sinclair, R.; Noerskov, J. K.; King, L. A.; Bajdich, M.; Jaramillo, T. F., Acidic

Oxygen Evolution Reaction Activity-Stability Relationships in Ru-Based Pyrochlores. *ACS Catal.* **2020,** *10* (20), 12182-12196.
